# ALDH1A1 promotes immune escape of tumor cells through ZBTB7B-glycolysis pathway

**DOI:** 10.1038/s41419-024-06943-9

**Published:** 2024-08-07

**Authors:** Mingyuan Wang, Taoli Wang, Jinjin Wang, Yuexin Yang, Xi Li, Huan Chen, Jingnan Liao

**Affiliations:** 1 Department of Geratic Surgery, Xiangya Hospital, Central South University, Changsha, Hunan China; 2 National Clinical Research Center for Geriatric Disorders, Xiangya Hospital, Central South University, Changsha, Hunan China; 3 Department of General Surgery, Xiangya Hospital, Central South University, Changsha, Hunan China; 4 Department of Pathology, the Affiliated Zhuzhou Hospital Xiangya Medical College, Central South University, Zhuzhou, Hunan China; 5 Department of Gynaecology, the Affiliated Zhuzhou Hospital Xiangya Medical College, Central South University, Zhuzhou, Hunan China; 6 Department of Oncology, the Affiliated Zhuzhou Hospital Xiangya Medical College, Central South University, Zhuzhou, Hunan China; 7 Hunan Provincial Key Laboratory of Regional Hereditary Birth Defects Prevention and Control, Changsha Hospital for Maternal & Child Health Care Affiliated to Hunan Normal University, Changsha, China

**Keywords:** Tumour immunology, Prognostic markers

## Abstract

The primary impediment to the success of immunotherapy lies in the immune evasion orchestrated by tumors, contributing to the suboptimal overall response rates observed. Despite this recognition, the intricacies of the underlying mechanisms remain incompletely understood. Through preliminary detection of clinical patient tissues, we have found that ALDH1A1 was a key gene for the prognosis of cancer patients and tumor glycolysis. In vitro experiments and tumor formation in nude mice suggested that targeting ALDH1A1 could inhibit tumor growth. Through further analysis of xenograft tumor models in immune-normal mice and flow cytometry, we found that deficiency in ALDH1A1 could promote immune system suppression of tumors in vivo. Specifically, RNA-seq analysis, combined with qPCR and western blot, identified the transcription factor ZBTB7B as downstream of ALDH1A1. The binding sites of the transcription factor ZBTB7B on the LDHA promoter region, which is responsible for regulating the rate-limiting enzyme gene LDHA in glycolysis, were determined using luciferase reporter gene detection and Chip-qPCR, respectively. In addition, the increased SUMOylation of ZBTB7B stabilized its transcriptional activity. Further in vivo and in vitro experiments confirmed that the combination of targeting ALDH1A1 and ZBTB7B with immune checkpoint inhibitors could synergistically inhibit tumors in vivo. Finally, after conducting additional verification of patient tissue and clinical data, we have confirmed the potential translational value of targeting ALDH1A1 and ZBTB7B for tumor immunotherapy. These results emphasize the potential translational significance of targeting ALDH1A1 and ZBTB7B in the realm of tumor immunotherapy. The convergence of ALDH1A1 inhibition and immune checkpoint blockade, particularly with PD-L1/PD-1 mAb, presents a compelling avenue for curtailing tumor immune escape.

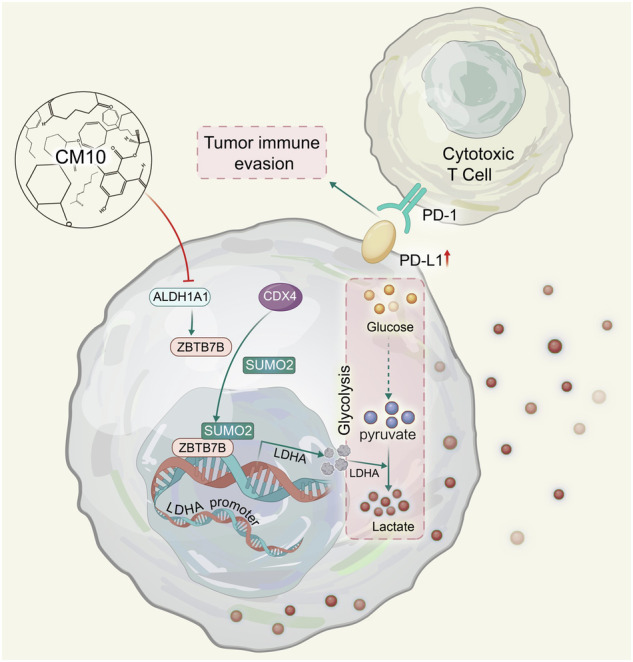

## Introduction

The advent of contemporary immunotherapy represents a beacon of hope for patients grappling with various malignancies, particularly non-small cell lung cancer (NSCLC), which is characterized by a paucity of treatment options. This breakthrough marks a significant milestone in the landscape of cancer therapeutics [1]. Immune checkpoint inhibitors (ICIs), typified by PD-1/PD-L1 inhibitors, have garnered considerable attention owing to their notable efficacy in treating a spectrum of solid tumors [2, 3]. However, the response rates to PD-L1/PD-1 blockade fall short of 40%, and certain patients manifest primary or secondary resistance to immunotherapy [4, 5]. The precise mechanisms underpinning tumor immune evasion remain elusive [6]. Therefore, comprehending the molecular intricacies governing the regulation of tumor immune escape assumes paramount importance, as it holds profound implications for refining the efficacy of anti-PD-L1/PD-1 therapy and shaping prognostic outcomes.

Accumulating empirical evidence accentuates the pivotal role of tumor cell glycolysis not only in sustaining tumorigenesis and cellular survival but also in orchestrating the intricate interplay between tumor cells and immune cells through the release of lactate [7]. Elevated concentrations of lactic acid can be internalized and metabolized as a crucial fuel substrate, thereby fostering tumor angiogenesis and facilitating tumor invasion and metastasis [8]. Moreover, heightened levels of lactic acid in the tumor microenvironment exert an immunosuppressive effect, activating the immune checkpoint PD-1 and instigating resistance against PD-L1/PD-1 therapy [9]. Recent investigations underscore the ability of the key rate-limiting enzyme HK2 in glycolysis to activate the NF-κB pathway, thereby promoting PD-L1 expression and facilitating immune evasion by tumors [10]. Consequently, modulating lactate accumulation by intervening in the glycolytic process of tumor cells emerges as a promising strategy to counteract tumor immune evasion.

Aldehyde dehydrogenase 1A1 (ALDH1A1), an NADP-dependent enzyme within the ALDH family, assumes a critical role in diverse physiological and toxicological processes [11, 12]. Identified as a marker for numerous tumor-initiating cells, ALDH1A1 contributes to maintaining the stemness of tumor cells, promoting tumor angiogenesis and metastasis, and playing a pivotal role in the development of resistance to anticancer drugs [13–15]. Importantly, ALDH1A1 has been implicated in lowering intracellular pH in breast cancer cells, suppressing antitumor immunity, and thereby fostering cancer progression [16]. This underscores ALDH1A1’s potential as a synergistic therapeutic target for immunotherapy, although the mechanistic understanding remains in its infancy.

The ZBTB family of transcription factors, distinguished by a BTB domain at the N-terminus and multiple zinc-finger domains at the C-terminus, assumes a pivotal role in a spectrum of physiological and pathological cellular events [17]. Existing literature posits ZBTB7A’s involvement in the regulation of gene transcription for key enzymes in cellular glycolysis [18]. Furthermore, antecedent research from our group has identified ZBTB34 and ZBTB41, members of the ZBTB family, as promoters of tumor proliferation and prognostic indicators [19, 20]. Nevertheless, the potential involvement of ZBTB proteins in the intricate process of tumor immune escape remains largely unexplored.

In this study, we elucidate that ZBTB7B, a member of the ZBTB family, functions as a transcriptional regulator of LDHA, the key rate-limiting enzyme controlling lactate production in tumor cells. Specifically, ALDH1A1 upregulates ZBTB7B in tumor cells, subsequently promoting the upregulation of LDHA. Enhanced SUMOylation of ZBTB7B further stabilizes its transcriptional activity. ALDH1A1, through the ZBTB7B-glycolysis pathway, instigates the excessive accumulation of lactic acid in the tumor microenvironment, culminating in the diminution of anti-tumor immune cell infiltration in the tumor tissue and, consequently, amplifying tumor immune escape. Our findings underscore the pivotal role of ALDH1A1 and ZBTB7B as critical regulators of lactate accumulation in tumors. Furthermore, these proteins emerge as potential predictive indicators for PD-L1/PD-1 therapeutic efficacy and promising targets for combination therapy.

## Materials and methods

### Experimental model and subject

Cell lines—referring to our previous methods [19–21], the human colon cancer cell line HT29, the human lung cancer cell line A549, the mouse colon cancer cell line CT26, and the mouse lung cancer cell line LLC were treated with 10% FBS (BI) cultured in DMEM medium (BI). Routine testing of all cell lines using the MycoAlert Mycoplasma Detection Kit (Lonza, Rockland) was negative.

Mice—Balb/c and Balb/c nude mice were purchased from Hunan SJA Laboratory Animal Co., Ltd, and bred in the Experimental Animal Center of Central South University. Mice used are female and specific pathogen free (SPF) grade and are usually injected subcutaneously at 6 weeks of age. In each treatment group, 5–7 mice were used to observe changes in tumor phenotype, and 5 mice were used to record the survival rate. The experimental mice were grouped according to the principle of random assignment. The blinded study was used, and the experimental implementer was blinded to the group of animals. This animal protocol was approved by the Ethics Committee of Xiangya Hospital (Central South University, Changsha, Hunan, China) (2021-XMSB-0200). All experiments strictly followed the guidelines for the investigation of pain in animal experiments to minimize animal suffering.

Clinical tissue samples and clinical data—Tumor tissues and corresponding follow-up data of patients with NSCLC (64 cases) and colon cancer (64 cases) who were hospitalized in Zhuzhou Hospital Affiliated to Xiangya School of Medicine, Central South University from 2015 to 2016 were collected. All tissues were stored at −80 °C. In addition, paraffin sections of nivolumab-responsive or non-responsive NSCLC patients hospitalized at Zhuzhou Hospital Affiliated to Xiangya School of Medicine, Central South University were also collected in this study. The study protocol has been approved by the Ethics Review Committee of Zhuzhou Hospital Affiliated to Xiangya School of Medicine, Central South University (ZZCHEC2021162-01). All tissue samples were collected in accordance with the informed consent policy and under the guidance of pathology experts without affecting the diagnosis and treatment of patients.

### Plasmids and vectors

For stable transfected cell lines, hairpin-carrying ALDH1A1 shRNA lentiviral vectors were purchased from Shanghai Genechem. Targeting sequences are described as follows: shALDH1A1 #H1: GCCAAATCATTCCTTGGAATT; shALDH1A1 #H2: CGGGCTAAAGAAGTATATCCTT; shAldh1a1 #M1: CCCAGTTCTTATCCAAGAATA; shAldh1a1 #M2: CGAGCTAAGAAATATGTTCTT; shZbtb7b #M1: CTGAACTATGAAGTCTTTGA A; shZbtb7b #M2: GAGATAGCTATAGTCCTCCTA.

For transient transfection reporter assays, all of these ALDH1A1-OE, Aldh1a1-OE, Zbtb7b-OE, Flag-Cdx4, HA-Zbtb7b, His-SUMO1/2/3, pGL3-CD274 promoter plasmids and pGL3-CD274-mut were Synthesized by Shanghai sangon biotech. Specific mutations of the putative binding sites were designed, and the sequences are listed in Fig. 4G.

For CRISPR sgRNA sequence: sgZbtb7b: Forward, 5′-caccgGGAGAAGATGGGGAGCCCCG-3′, Reverse, 5′-aaacCGGGGCTCCCCATCTTCTCCc-3′.

### Determination of glycolytic indicators

Glucose Uptake Colorimetric Assay Kit, Pyruvate Colorimetric Assay Kit, Lactate Assay Kit, ATP Colorimetric Assay Kit were purchased from Biovision (Biovision, USA) and used to measure glucose uptake, pyruvate, lactate, and ATP level. For specific operation methods, refer to the manufacturer’s instruction manual.

The ECAR and OCR were determined using the Seahorse Bioscience XF96 Extracellular Flux Analyzer (Seahorse Bioscience, USA). Experiments were performed according to the manufacturer’s instructions. ECAR and OCR were measured using the Hippocampus XF Glycolytic Stress Test Kit and the Hippocampus XF Cell Mitotic Stress Test Kit (Agilent Technologies), respectively.

### Western blot analyses

Referring to our previous research method [22], cells were lysed in RIPA lysis buffer (Beyotime, China) supplemented with protease inhibitors (Selleck, USA). Protein concentration was measured using a BCA reagent (Beyotime, China) meter. Equal amounts of proteins were separated by SDS-PAGE and immunoblotted with different antibodies. Immunoblots were detected using a gel image analysis system (Bio-Rad, USA). The gray value of the bands was analyzed by ImageJ software, and the protein expression was quantitatively analyzed. The primary antibodies used were anti-ALDH1A1 (Abcam, USA), anti-LDHA (Abcam, USA), anti-β-actin (Cell Signaling Technology, USA), anti-AKT and anti-p-AKT (Abcam, USA), anti-mTOR and anti-p-S6K (Abcam, USA), anti-ZBTB7B (Abcam, USA), anti-PD-L1 (Abcam, USA), anti-Xbp1 (Abcam, USA), anti-Gata4 (Abcam, USA), and anti-Flag/HA/His tag (Abcam, USA).

### RNA extraction and qRT-PCR

RNA was extracted using the Trizol method. The RNA reverse transcription procedure was performed according to the instructions of the TaKaRa reverse transcription kit (TaKaRa, China). The qPCR primer sequences of related genes were designed and synthesized by Shanghai Shenggong Bioengineering Co., Ltd. All genes have been compared to Actb as an internal reference gene. The primer sequences are shown in Supplementary Table S1.

### Dual luciferase reporter assay

Briefly, the target DNA fragment was obtained by extracting CT26 cell DNA and PCR technique, and the fragment was inserted into the pGL-3 reporter gene vector. The Ldha promoter-pGL-3 recombinant plasmid and the Zbtb7b overexpression vector were co-transfected into WT CT26 cells. Subsequently, a microplate reader was used to detect the intensity of the luciferase reaction in the dark. Bioinformatics analysis predicted the possible binding site of Zbtb7b in the Ldha promoter region. A site-directed mutagenesis kit (Biotechnology, China) was used to mutate multiple adjacent bases at the predicted binding site to construct a reporter gene for site-directed mutagenesis of the Ldha promoter. Binding of Zbtb7b to target DNA was determined by comparing the ratio of the response intensities of firefly luciferase and Renilla luciferase.

### ChIP analysis of Zbtb7b and Ldha promoter region binding

After DNA immobilization, the long DNA strands are broken into DNA fragments of 200–1000 base pairs. Adjust the concentration of the system, adding Zbtb7b antibody and IgG antibody. Then, unbound DNA fragments are discarded and solution buffer is added to elute the antibody from the complex. Target DNA fragments were detected by PCR.

### Generation of CRISPR-edited cells

To generate Zbtb7b-KO cell lines, specific guide RNAs (sgRNAs) were designed using the CRISPR MIT tool (Zhang Lab, MIT). Briefly, the sequence of the sgRNA targeting Zbtb7b was cloned into the LentiCRISPR2 vector. The lentiviral construct was transiently transfected into HEK293T cells using PolyJet transfection reagent (SignaGen, USA) and placed in 1 μg/mL puromycin (Sigma-Aldrich, USA) to select successfully transfected cells. 48 hours after selection, Zbtb7b-deficient monoclonals were picked. Characterization was performed by WB to assess the reduction of Zbtb7b expression and gene editing of Zbtb7b was confirmed by sequencing.

### T-cell co-culture model

CD8+ T cells were sorted from spleens of BABL/c mice using flow cytometry and placed on supplemented IL-2 (1000 U/mL). On day 1, cells were seeded in 6-well plates at a density of 2 × 10^6^ per well and treated with mouse IFN-γ (1000 U/mL). On day 2, anti-CD3 antibody (50 ng/mL) and IL-2 (1000 U/mL) were added to the medium to promote T cell activation. Stimulated CD8+ T cells were then harvested and co-cultured with CT26 cells at a ratio of 10:1 for 24 h. T cells and cell debris were removed by washing with PBS, then viable cancer cells were quantified by a spectrometer at OD (570 nm), followed by crystal violet staining.

### Flow cytometry analysis

In this study, all above-mentioned flow cytometry antibodies and reagents were purchased from BioLegend, San Diego, CA, USA. For mouse samples, single-cell suspensions of CT26 xenografts were obtained by quick and gentle dissection, physical trituration, and filtration. After removing dead cells, cells were stained for 20 min with APC-CD3 (100236), PECY17-CD8α (100722), BV421-PD1 (135218), BV785-TIM3 (119725) and APC-PD-L1 (124312). After fixation and permeabilization by True-Nuclear Transcription factor Buffer Set, intracellular Gzmb was stained using FITC-Gzmb antibody. Stained cells were analyzed by FACS Dxp AthenaTM (Cytek, USA). Data were further analyzed by Flow Jo 10.0 software.

### Immunohistochemical and Immunofluorescence

Each tissue sample was stained with the indicated primary antibody and biotin-conjugated secondary antibody, followed by incubation with avidin-biotin-peroxidase complex. Visualization of target proteins was performed by diaminobenzidine (DAB), where the presence of brown color indicates the expression of target molecules. To quantify these immunohistochemical results, images were further processed by 3D HISTECH Quantitative Center 2.1 software. The staining intensity of the target protein and the percentage of tumor cell area were analyzed by software.

For immunofluorescence, 4 μm paraffin sections of patient samples were bake-deparaffinized. Antigens were recovered in EDTA antigen retrieval buffer (pH 8.0) and held at subboiling temperature for 8 min, resting for 8 min, and then another subboiling temperature for 7 min. After autofluorescence quenching, samples were blocked with 3% BSA, PBS and 0.25% Triton X-100 for 1 h at room temperature. Incubate the primary antibody in blocking solution overnight at 4 °C and the next day at room temperature for 30 min. After extensive washing in PBS-0.25% Triton X-100, the secondary antibody was added to the blocking solution and incubated for 2 h. Each primary antibody was stained separately and antigen was retrieved again by microwave in EDTA antigen retrieval buffer (pH 8.0) between each staining interval. After extensive washing in PBS-0.25% Triton X-100, cover slips with anti-fade mounting medium. Then incubated with DAPI solution for 10 min at room temperature and store in a dark place. Detect and capture images by fluorescence microscopy.

### RNA-seq analysis

RNA-seq library construction was completed by Huada Gene Company (Shenzhen, China). Raw data were processed using the CASAVA V1.6 software package. Quality control of each sample was completed using FASTQC V0.11.5. HISAT47 aligns clean reads to the human reference genome hg19. Gene expression was calculated using RSEM48 and differential expression was determined by an algorithm developed at BGI. Differentially expressed genes (DEGs) with |log2(fold change)| > 1 and FDR < 0.001 were considered significant. Gene set enrichment analysis (GSEA) was performed using Xiantao Academic Online Tools (https://www.xiantao.love/products). All data, including sequencing reads and expression matrices, have been deposited in NCBI’s Gene Expression Omnibus Database (http://www.ncbi.nlm.nih.gov/geo/). All datasets generated in this study can be accessed under a GEO superseries with accession number GSE225822 and GSE269935.

### ALDH activity assay

The ALDH Activity Assay Kit (Abcam, ab155893) was utilized for the quantification of ALDH activity in HT29 cells treated with CM10 at a concentration of 10 μM for a duration of 2 h. In the experimental procedure, NADH standards were added to a 96-well plate, and the volume was adjusted to 50 μl per well with the ALDH Assay Buffer. The cells were then mixed with the ALDH assay buffer to remove nuclei and insoluble substances. Subsequently, the plate was incubated at room temperature for 20–60 min, and the absorbance is measured at 450 nm.

### Extracellular lactate measurement

ALDH1A1 (Abcam, ab89491) solutions at various concentrations were prepared and added to the supernatants of HT29 and A549 cells for 10 min. The lactate concentration in the supernatants was measured using a human lactate ELISA kit (Abcam, ab65330). Briefly, 10 μl of each sample was mixed with 40 μl of lactate assay buffer in a 96-well plate, and the standards were prepared using the kit’s assay buffer. The reaction mixture was added to each well, incubated for 30 min at room temperature away from light, and the absorbance was read at 570 nm.

### Immunoprecipitation assay

CT26 cells were seeded on 10 cm plates. After 24 hours, cells were harvested and incubated in lysis buffer containing protease inhibitor cocktail (40 mM HEPES, 120 mM NaCl, 10 mM sodium pyrophosphate, 10 mL sodium glycerophosphate, 1 mM ethylenediaminetetraacetic acid (EDTA), 50 mM sodium fluoride, 0.5 mM sodium orthovanadate, 1% Triton X-100), and then rotated at 4 °C for 1 h to dissolve the protein. Soluble protein was collected and immunoprecipitated overnight with the antibodies. Add Protein-A/G agarose beads to protein lysates and incubate overnight in the refrigerator. Beads were centrifuged and washed at least three times with lysis buffer. After the supernatant was discarded, the precipitate was added to 25 μl of 2 × loading buffer, the sample was boiled and centrifuged, and the supernatant liquid was absorbed to verify the interaction between the target proteins by western blotting.

For the Ni^2+^ bead pull-down assay, Ni^2+^-NTA agarose was used to precipitate His-tagged SUMO1/2/3 from cell lysates. Resolved by 8% SDS-PAGE and processed for immunoblotting.

### Mouse tumor model

For the immunodeficient mouse model, CT26 cells were injected subcutaneously into Balb/c nude mice in 100 μL of culture medium. Tumor growth was measured using digital calipers, and tumor size was recorded. For the immunocompetent mouse model, the indicated tumor cells were injected subcutaneously into Balb/c mice. The initial dose of cells injected subcutaneously was 5 × 10^6^ cells. After the experiment was over, the tumor was collected and the tumor weight was measured. For live mice, subcutaneous tumors were measured using bioluminescent imaging after anesthesia with isoflurane.

### Statistical analysis

Data are expressed as mean ± S.D. Statistical analysis was performed by Student’s *t* test when only two value sets were compared. Two-way ANOVA was used when the data involved three or more groups. Survival was calculated and analyzed using the Kaplan–Meier method, and the Gehan Breslow-Wilcoxon test was used to test for differences between survival curves. For correlation analysis, correlation coefficients r and *p* values were calculated based on Spearman’s rank correlation method. *P* < 0.05, *P* < 0.01 or *P* < 0.001 were considered to be statistically significant, indicated by *, ** or ***, respectively.

## Results

### ALDH1A1 deficiency suppresses tumor glycolysis and inhibits tumor growth in immunodeficient mice

**Fig. 1:**
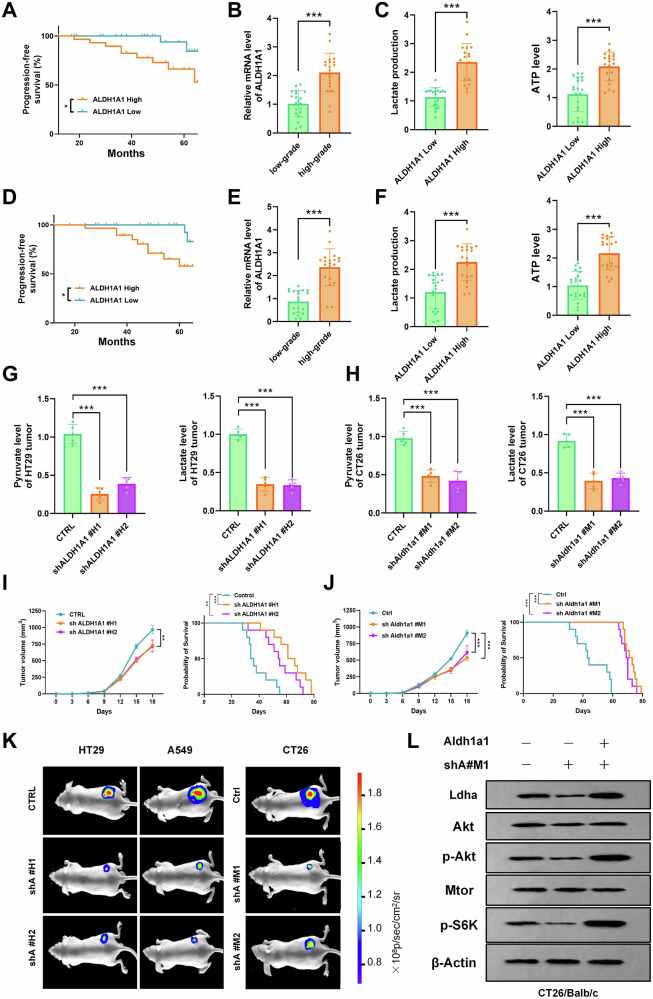
Genetic deficiency of ALDH1A1 reduced levels of tumor glycolysis and was associated with tumor growth suppression in an immunodeficient mouse model. **A** Kaplan–Meier survival curves of PFS in groups with high (32 cases) and low (32 cases) ALDH1A1 expression in NSCLC patients. **B** Histograms of ALDH1A1 expression in low-grade (highly differentiated, 20 cases) and high-grade (poorly differentiated, 20 cases) NSCLC tumors. **C** Histograms of glycolysis markers—lactate and ATP in NSCLC tumor tissues of the ALDH1A1 high (20 cases) and low (20 cases) expression group. **D** Kaplan–Meier survival curve of PFS in groups with high (32 cases) and low (32 cases) expression of ALDH1A1 in colon cancer patients. **E** Histogram of ALDH1A1 expression levels in low-grade (20 cases) and high-grade (20 cases) tumor tissues of patients with colon cancer. **F** Histograms of glycolysis markers—lactate and ATP in the colon cancer tissues of the ALDH1A1 high (20 cases) and low (20 cases) expression group. **G** Histograms of key products of glycolysis pyruvate and lactate in confounding negative control (CTRL) and two shALDH1A1 (#H1 and #H2) transfected HT29 xenografts in BALB/c nude mice. **H** Histograms of the key products of glycolysis pyruvate and lactate in confounding negative control (Ctrl) and two shAldh1a1 (#M1 and #M2) transfected CT26 xenografts in BALB/c nude mice. **I** Tumor growth curves of confounding negative control (CTRL) and two shALDH1A1 (#H1 and #H2) transfected HT29 xenografts in BALB/c nude mice and Kaplan–Meier survival curves of these mice (*n* = 10). **J** Tumor growth curves of confounding negative control (Ctrl) and two shAldh1a1 (#M1 and #M2) transfected CT26 xenografts in BALB/c nude mice and Kaplan–Meier survival curves of these mice (*n* = 10). **K** Cell lines from different sources (human intestinal cancer cell HT29, human lung cancer cell A549, and murine intestinal cancer cell CT26) were transfected with a mixed negative control (CTRL/Ctrl) and two shALDH1A1 (#H1 and #H2; #M1 and #M2) In vivo imaging photos of xenograft tumor models constructed in BALB/c nude mice. **L** Western blot analysis of LDHA/Akt/p-Akt/Mtor/p-S6K protein expression levels after shAldh1a1 #M1 knockdown and compensation experiments in CT26 cells (with β-Actin as internal reference). P.S. We specified that grade 1 was a low-grade tumor and grades 2–3 was a high-grade tumor. Results were presented as mean ± S.D.; ns not significant; *P < 0.05, **P < 0.01, ***P < 0.001.

The acidification of the tumor microenvironment is intricately linked to the specific glucose metabolism pattern of the tumor [23]. Tumor glycolysis serves as an energy source crucial for sustaining tumor proliferation [24]. Additionally, glycolysis contributes to the excessive accumulation of lactic acid in the tumor microenvironment, creating a highly acidic milieu conducive to tumor progression [25]. Previous study has indicated that ALDH1A1 plays a role in lowering intracellular pH, supporting the maintenance of an acidic tumor microenvironment [16]. However, its specific role in tumors and its involvement in tumor cell glycolysis remain unclear. We collected tumor tissues from patients who underwent surgery for NSCLC and colon cancer, respectively. According to the immunohistochemistry of ALDH1A1 in the tissue sections, the samples were divided into two groups: the high expression group and the low expression group. The progression-free survival (PFS) analysis revealed that the ALDH1A1 high expression group exhibited significantly lower PFS than the low expression group, both in lung cancer and colon cancer (Fig. 1A, D). Further analysis demonstrated elevated ALDH1A1 expression in pathologically diagnosed high-grade (poorly differentiated) tumor tissues (Fig. 1B, E). Additionally, the ALDH1A1 high expression group exhibited elevated levels of glycolysis markers, including lactate and ATP, in tumor tissues (Fig. 1C, F). These findings underscore ALDH1A1’s pivotal role in tumor progression and its strong association with tumor glycolysis.

To investigate the impact of ALDH1A1 on tumor cell glycolysis in vitro, specific small hairpin RNA (shRNA) targeting human and mouse ALDH1A1 was generated and validated in three distinct tumor cell lines (Supplementary Fig. S1A–C). Knockdown of ALDH1A1 in human HT29 and A549 cells, as well as Aldh1a1 in murine CT26 cells, significantly impeded cell proliferation, with this inhibition intensifying over time (Supplementary Fig. S2A–D). Utilizing glucose uptake, pyruvate and lactate levels, ATP production, extracellular acidification rate (ECAR), and oxygen consumption rate (OCR) as specific glycolysis markers [26, 27], it was observed that ALDH1A1 knockdown significantly reduced pyruvate and lactate levels in HT29 cells (Fig. 1G) and exhibited a similar trend in murine CT26 cells (Fig. 1H).

Subsequently, HT29 and CT26 cells were inoculated into immunodeficient (BALB/c nude) mice for in vivo studies. Compared with the control group, the tumor volume in the shALDH1A1 or shAldh1a1-transfected groups was significantly diminished (Fig. 1I, J). Moreover, the survival time was prolonged in the shALDH1A1 or shAldh1a1 groups compared to the control group (Fig. 1I, J). In vivo imaging technology corroborated these findings, showing significantly restricted tumor growth in the transfected shALDH1A1 or shAldh1a1 groups in nude mouse xenograft tumor models constructed using HT29, A549, and CT26 cells (Fig. 1K).

Glycolysis, as a series of chain reactions, results in the breakdown of glucose into pyruvate and ultimately into lactic acid, concomitant with ATP production [28]. Through ALDH1A1 knockdown and compensation experiments, it was established that ALDH1A1 serves as an upstream driver of glycolytic effects in tumor cells (Supplementary Figs. S3–S5). As the key rate-limiting enzyme for lactate production, lactate dehydrogenase A (LDHA) is essential for synthesizing lactate (Supplementary Fig. S6A). Knocking down ALDH1A1 inhibited LDHA expression in HT29, A549, and CT26 cells (Fig. 1L and Supplementary Fig. S6B, C). Subsequently, the impact of LDHA expression on tumor growth was investigated by knocking down Ldha in CT26 murine tumor cells and inoculating them into immunodeficient (BALB/c nude) mice for in vivo studies, revealing significantly restricted tumor growth in the shLdha group (Supplementary Fig. S6D). This suggests that, in the absence of immunity, ALDH1A1 influences tumor growth by regulating LDHA.

The Akt/mTOR signaling pathway is an important regulatory signaling pathway for tumor proliferation and invasion [29]. In the present study, we sought to demonstrate the pro-tumor effect of ALDH1A1 by examining the Akt/mTOR signaling pathway, thereby demonstrating the potential feasibility of targeting ALDH1A1 for cancer therapy. Our study found that, ALDH1A1 knockdown decreased p-Akt and p-S6K expression downstream of mTOR, whereas overexpressing ALDH1A1 restored p-Akt and p-S6K (Fig. 1L and Supplementary Fig. S7). These results suggest that ALDH1A1 may be an important upstream signal for Akt/mTOR and regulate tumor proliferation in the absence of immunity, which is also consistent with previous study [30].

### ALDH1A1 deficiency enhances immune-mediated tumor confinement in vivo

**Fig. 2:**
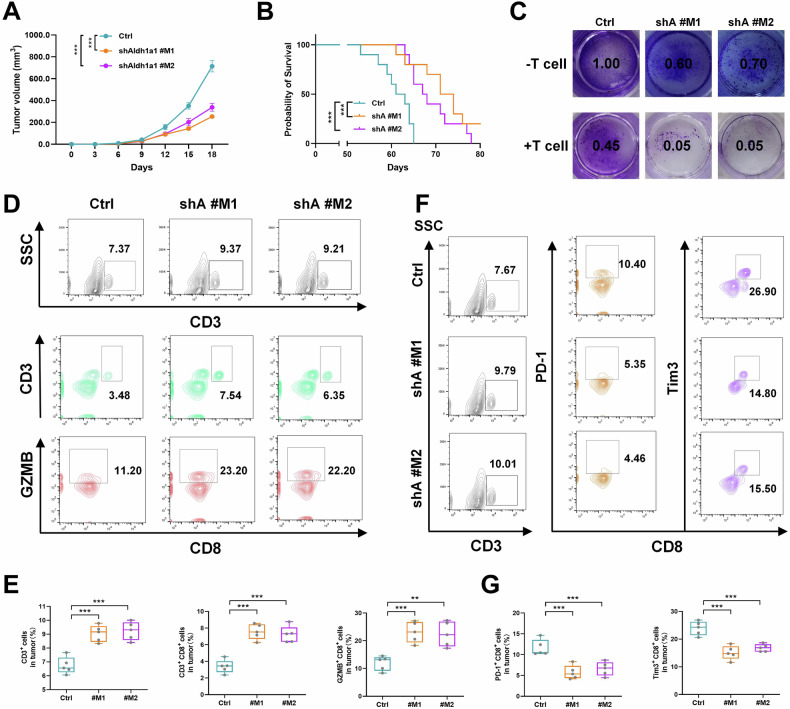
ALDH1A1 deficiency promoted tumor suppression by the immune system in vivo. Tumor growth curves of (**A**) confounding negative control (Ctrl) and two shAldh1a1 (#M1 and #M2) transfected CT26 xenografts in BALB/c mice and (**B**) Kaplan–Meier survival curves. **C** T cell killing assay in CT26 cells transfected with shAldh1a1 (#M1 and #M2) or control (Ctrl). **D**, **E** CD3+ and CD3+ CD8+, GZMB+ CD8+ TILs and quantitative statistics of BALB/c mouse tumor tissue in shAldh1a1 (#M1 and #M2) and control group (Ctrl) by flow cytometry. **F**, **G** CD3+ and CD8+ PD-1+, CD8+ Tim3+ TILs and quantitative statistics of BALB/c mouse tumor tissue in shAldh1a1 (#M1 and #M2) and control group (Ctrl) by flow cytometry. Results were presented as mean ± S.D., *n* = 3–5. ns not significant; *P < 0.05, **P < 0.01, ***P < 0.001.

To assess the influence of the intact immune system on the anti-tumor effects of shAldh1a1, CT26 mouse colon cancer cells were injected into immunocompetent BALB/c mice. Notably, the tumor size in the shAldh1a1 group exhibited a significant reduction, and the survival time of tumor-bearing mice was markedly prolonged (Fig. 2A, B). Similarly, in immunoactive BALB/c mice, Ldha deficiency also substantially decelerated tumor growth and extended the survival time of tumor-bearing mice (Supplementary Fig. S8).

Furthermore, an exploration into whether ALDH1A1 impacts the anti-tumor efficacy of the immune system was undertaken. T cell-mediated cancer cell killing experiments revealed that Aldh1a1 downregulation in CT26 cells rendered them more susceptible to T cell-mediated killing (Fig. 2C). Flow cytometric analysis of immune cell proportions in mouse tumor tissues showed a significant increase in infiltrating CD3+ and CD3+ CD8+ T cells in the shAldh1a1 group compared to the control group. Notably, killer CD8+ T cells (GZMB+ CD8+) were also substantially elevated in the shAldh1a1 group (Fig. 2D, E). Additionally, a significant decrease in PD-1+ and Tim3+ infiltrating CD8+ T cells was observed in the shAldh1a1 group (Fig. 2F, G). These findings suggest that Aldh1a1 downregulation enhances the immune system’s tumor-killing efficacy and mitigates immune escape in mice with intact immune systems.

To elucidate the relationship between the anti-tumor effect of Aldh1a1 knockdown and CD8+ T cells, shAldh1a1 cells were injected into immunocompetent mice, and CD8α monoclonal neutralizing antibody (mAb) or immunoglobulin G (IgG) isotype Ctrl (IgG2b) was administered to the mice (Supplementary Fig. S9A). Consistent with previous studies [31], mice inoculated with CT26 cells and treated with CD8α mAb exhibited CD8+ T cell suppression and a significant increase in tumor burden compared to those treated with IgG2b (Supplementary Fig. S9B–D). This underscores the critical role of CD8+ T cells in maintaining the immune system’s antitumor effect. Depletion of CD8+ T cells was found to compromise shAldh1a1-mediated tumor suppression, aligning with prior literature reports [32].

### ALDH1A1 facilitates tumor immune escape by inducing glycolysis in vivo

**Fig. 3:**
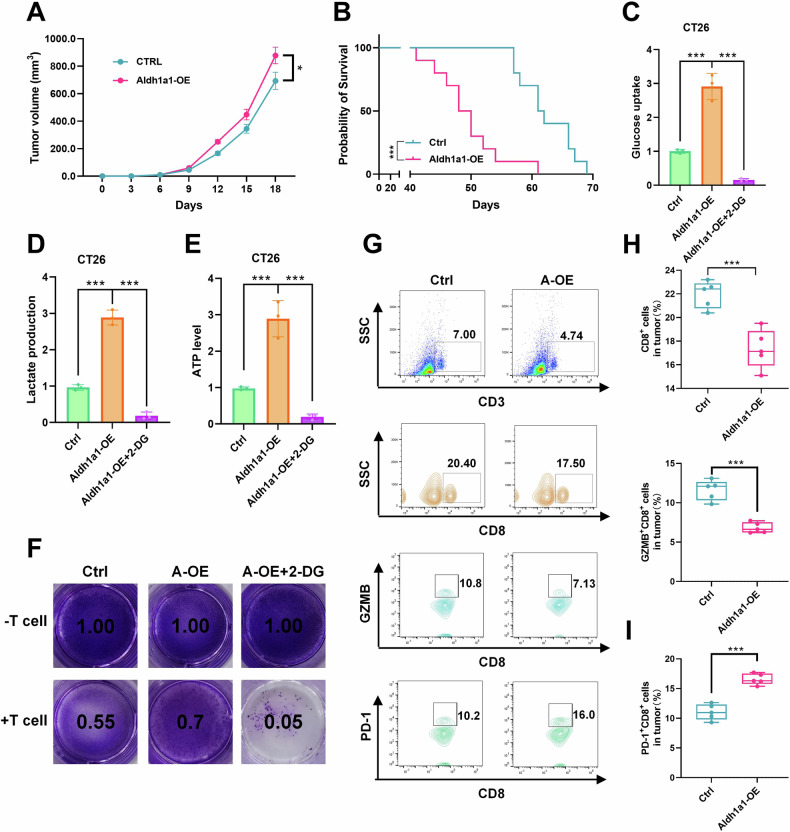
ALDH1A1 mediated tumor immune escape by regulating glycolysis in vivo. Tumor growth curves (**A**) of confounding negative control (Ctrl) and Aldh1a1-OE-transfected CT26 xenografts in BALB/c mice and Kaplan–Meier survival curves (**B**) of these mice. Histograms of key markers of glycolysis glucose uptake (**C**), lactate production (**D**) and ATP level (**E**) after Aldh1a1-OE overexpression and 2-DG inhibition of glycolysis in CT26 cells. **F** T cell killing assay in CT26 cells after transfection with sAldh1a1-OE overexpression and 2-DG inhibition of glycolysis. **G**–**I** TILs and quantitative statistics of CD3+ and CD8+ GZMB+, CD8+ PD-1+ TILs of BALB/c mouse tumor tissue in Aldh1a1-OE and control group (Ctrl) by flow cytometry. Results were presented as mean ± S.D., *n* = 3–5. ns not significant; *P < 0.05, **P < 0.01, ***P < 0.001.

To investigate whether ALDH1A1 contributes to tumor immune evasion by elevating the glycolytic activity of tumor cells within an intact immune system, a xenograft model was established in normal immune BALB/c mice using CT26 cells that overexpress Aldh1a1. Notably, the overexpression of Aldh1a1 promoted tumor growth and decreased the overall survival of mice with tumors (Fig. 3A, B). Furthermore, Aldh1a1 overexpression heightened glucose uptake, lactate production, and ATP content in CT26 cells (Fig. 3C–E). Treatment with 2-Deoxy-D-glucose (2-DG), a glucose analog known to inhibit glycolysis by targeting hexokinase [33], effectively blocked the Aldh1a1-induced increases in the aforementioned indicators (Fig. 3C–E). The same trend also appeared in HT29 and A549 cells (Supplementary Fig. S10A, C). Not only that, after adding ALDH1A1, the lactate content in the supernatant of cells increased with the increase in the dose of ALDH1A1 (Supplementary Fig. S10B, D). In normal immune mouse tumor models, upregulation of Ldha can also promote tumor growth and reduce the overall survival of tumor mice (Supplementary Fig. S11). These findings collectively imply that ALDH1A1 upregulates glycolysis in tumor cells, thereby promoting tumor growth.

In vitro experiments utilizing stimulated T cells demonstrated that the overexpression of Aldh1a1 conferred enhanced resistance of CT26 cells to killer T cells. Notably, inhibiting glycolysis significantly augmented the anti-tumor capacity of killer T cells (Fig. 3F). Flow cytometric analysis revealed that Aldh1a1 overexpression led to a suppression in the ratio of infiltrating CD8+ T cells and GZMB+ CD8+ T cells in tumors (Fig. 3G, H). Furthermore, a conspicuous increase in the infiltration of suppressive CD8+ T cells (PD-1+) was observed (Fig. 3G, I). These results collectively underscore the capacity of Aldh1a1 to promote tumor glycolysis and enhance immune evasion.

To explore whether Aldh1a1 knockdown (KD) could synergize with immunotherapy, shAldh1a1 cells were inoculated into immunocompetent mice, and subsequent treatment involved PD-L1 monoclonal antibody (mAb) or IgG isotype control (IgG2a) administration (Supplementary Fig. S12A). In CT26 tumor-bearing mice, PD-L1 mAb treatment significantly increased the infiltration of killer CD8+ T (GZMB) cells compared to IgG2a (Supplementary Fig. S12B, C) and markedly reduced tumor burden (Supplementary Fig. S12D, E). These findings suggest that inhibiting Aldh1a1 may serve as a potential synergistic therapeutic approach for PD-L1 mAb immunotherapy.

### ZBTB7B functions as a downstream target of ALDH1A1, regulating LDHA transcription in tumor cells

**Fig. 4:**
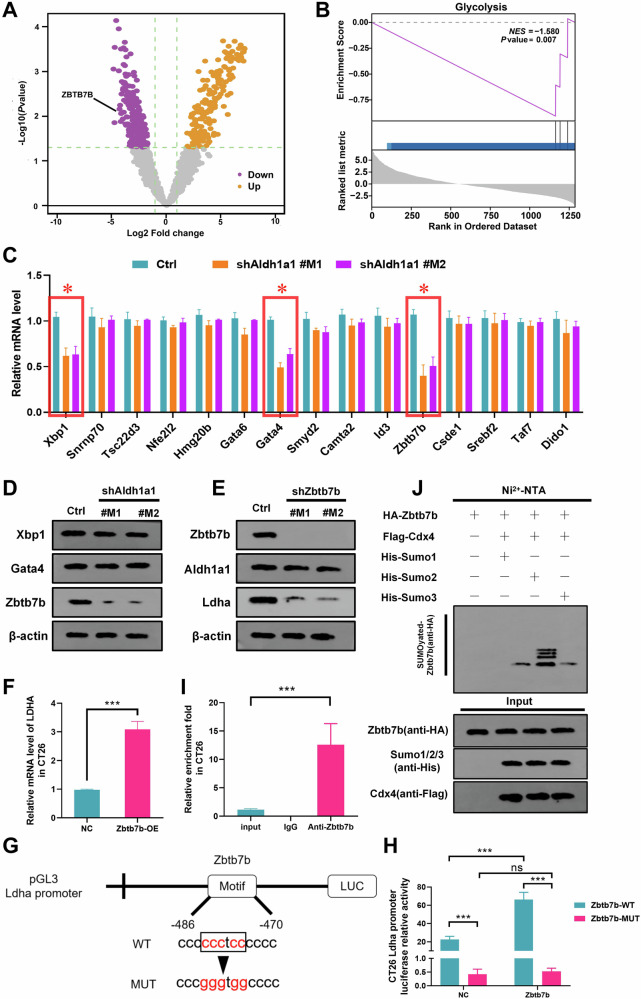
The ALDH1A1-ZBTB7B signaling axis transcriptionally regulated LDHA levels in tumor cells. **A** Volcano plot of differentially expressed genes in the HT29 cell line transfected with shALDH1A1 and the control group (CTRL) (*P* < 0.05). The |log2 (fold change)| of −1, and 1. Purple indicated low expression genes, yellow were highly expressed genes. **B** GSEA enrichment analysis of the differential genes in Fig. 4A on the glycolysis pathway. **C** In CT26 cells, qPCR of shAldh1a1 (#M1 and #M2) versus the control group (Ctrl) was used to verify the candidate genes with significant differences in RNA-seq. **D** In CT26 cells, candidate genes with significant differences in qPCR were verified by western blot of shAldh1a1 (#M1 and #M2) and control group (Ctrl). **E** In CT26 cells, the expression differences of Aldh1a1 and Ldha were detected by Western blot of shZbtb7b (#M1 and #M2) and the control group (Ctrl). **F** Histogram of Ldha mRNA expression changes after Zbtb7b overexpression (-OE) detected by qPCR. **G** Schematic diagram of the relative position of Ldha promoter full-length (FL) and Zbtb7b binding. WT is the wild-type sequence, and MUT is the mutant sequence. **H** Histogram of the Zbtb7b binding site wild-type and mutant luciferase reporter assays. **I** Analysis of the chromatin sequence of the Ldha promoter region pulled down by Zbtb7b antibody by ChIP–qPCR. **J** Western blot detection of the main SUMO modification types of Zbtb7b in CT26 cells. Data were mean ± S.D. of three independent experiments. (ns not significant; *P < 0.05, **P < 0.01, ***P < 0.001).

To unveil the downstream signaling pathways implicated in ALDH1A1-mediated tumor immune escape, we performed RNA-sequencing and differential analysis in shALDH1A1 HT29 cells and CTRL cells (Fig. 4A). Gene Set Enrichment Analysis (GSEA) demonstrated a significant downregulation of signals related to glycolysis in the shALDH1A1 group (Fig. 4B). Among the top 15 genes with reduced expression, Xbp1, Gata4, and Zbtb7b displayed consistent expression trends in CT26 cells based on qPCR analysis (Fig. 4C). Western blot analysis confirmed the presence of the transcription factor ZBTB7B in all candidate genes (Fig. 4D). Mouse-derived shRNA targeting Zbtb7b was constructed, and its inhibition significantly reduced LDHA protein expression without affecting Aldh1a1 expression (Fig. 4E). Conversely, Zbtb7b overexpression markedly upregulated the mRNA expression of LDHA (Fig. 4F). Through dual intervention of Aldh1a1 and Zbtb7b knockdown compensation experiments, we conclusively identified Zbtb7b as the downstream target of Aldh1a1. Furthermore, we demonstrated that Zbtb7b serves as a transcriptional regulator of LDHA (Supplementary Fig. S13).

To ascertain if LDHA is a direct target gene of Zbtb7b, bioinformatics screening identified a potential Zbtb7b binding site in the mouse Ldha gene promoter region, based on the Zbtb7b motif (Fig. 4G). Promoter constructs with mutations in this region were generated, leading to defects in Zbtb7b binding. A dual-luciferase assay using CT26 cell lines with WT or Mut promoter elements demonstrated that the promoter mutation decreased Ldha expression in both the control and Zbtb7b groups (Fig. 4H). Furthermore, exogenous expression of Zbtb7b in the CT26 cell line, assessed through a chromatin immunoprecipitation assay, revealed significant enrichment of the Ldha promoter region by Zbtb7b (Fig. 4I). Collectively, these findings provide evidence that Zbtb7b positively regulates Ldha mRNA expression in CT26 cells.

SUMOylation, a crucial post-translational modification, exerts diverse regulatory effects on protein functions. Using the Ni2 + -NTA agarose bead pull-down assay, it was observed that Zbtb7b undergoes SUMO2-based ubiquitin-like modification, facilitated by the E3 ligase Cbx4 (Fig. 4J). Additionally, stable transfection of the Aldh1a1 overexpression plasmid into CT26 cells enhanced the SUMOylation of Zbtb7b, as demonstrated by the Ni2 + -NTA agarose bead pull-down test (Supplementary Fig. S14A). Bioinformatics analysis and construction of the K508R site mutant, a potential SUMO modification site of Zbtb7b, revealed that the SUMOylation site is located at Lysine 508 (Supplementary Fig. S14B). These results further underscore Aldh1a1 as a key upstream regulator of Zbtb7b, enhancing its transcriptional activity by upregulating SUMOylation.

### Zbtb7b deficiency enhances the anti-tumor effect of PD-L1 mAb in mice

**Fig. 5:**
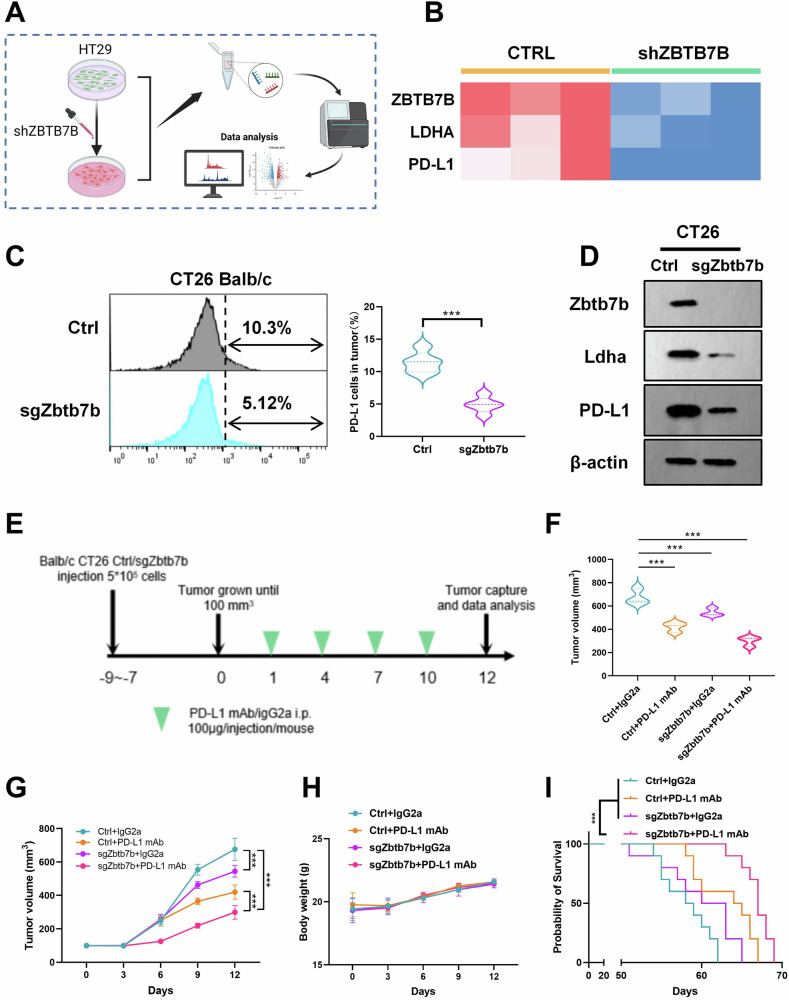
Zbtb7b deficiency enhanced the antitumor effect of PD-L1 mAb in vivo. **A** Schematic diagram of the construction of RNA-sequencing in ZBTB7B knockdown HT29 cell line and CTRL cells. **B** RNA-seq showed heat maps of ZBTB7B, LDHA, and PD-L1 expression between shZBTB7B cells and control cells. **C** Flow cytometry analysis of PD-L1 expression and quantitative statistics in CT26 cell lines transfected with sgZbtb7b or Ctrl sgRNA. **D** Western blot detection of the protein expressions of Zbtb7b, Ldha, and PD-L1 in CT26 cell lines transfected with sgZbtb7b or Ctrl sgRNA. **E**–**I** BALB/c mice implanted with 5 × 10^5^ sgZbtb7b CT26 or Ctrl cells and received PD-L1 mAb treatment or IgG isotype control (IgG2a). **E** Scheme schematic of a treatment timeline. **F** Volume statistics of CT26 tumors harvested after euthanasia of BALB/c xenografted mice. **G** Line graph of tumor volume over time. **H** Mouse body weight was measured every 3 days. **I** Kaplan–Meier survival curves for each group. Results were presented as mean ± S.D., *n* = 3–5. ns not significant; *P < 0.05, **P < 0.01, ***P < 0.001.

A ZBTB7B knockdown HT29 cell line and a control (CTRL) cell line were constructed for RNA sequencing (Fig. 5A). RNA-seq re-validation confirmed that ZBTB7B knockdown resulted in reduced expression levels of LDHA and PD-L1 in tumor cells (Fig. 5B), establishing LDHA and PD-L1 as downstream effectors of ZBTB7B. To assess the anti-tumor effect of Zbtb7b in the normal immune system, Zbtb7b-deficient (sgZbtb7b) CT26 and LLC cell lines were created, and in vivo experiments were conducted using normal immune BALB/c mice. Flow cytometric sorting revealed a significant reduction in the proportion of PD-L1 tumor cells in both cell groups treated with sgZbtb7b (Fig. 5C and Supplementary Fig. S15A). Additionally, sgZbtb7b significantly downregulated the protein expression of Ldha and PD-L1 in both cell types (Fig. 5D and Supplementary Fig. S15B).

To investigate whether sgZbtb7b enhances the anti-tumor efficacy of PD-L1 mAb, sgZbtb7b CT26 and LLC cells were inoculated into immunocompetent mice and treated with PD-L1 mAb or IgG isotype control (IgG2a) (Fig. 5E and Supplementary Fig. S16A). In mice inoculated with CT26 cells, both sgZbtb7b and PD-L1 mAb treatments alone significantly reduced tumor burden. Notably, when the tumor model of sgZbtb7b cells was treated with PD-L1 mAb, the tumor burden was minimal (Fig. 5F, G). Similar trends were observed in LLC cells (Supplementary Fig. S16B, C). Importantly, the combination of sgZbtb7b and PD-L1 mAb did not cause significant damage to the body weight of mice but did prolong the survival time of tumor-bearing mice (Fig. 5H, I and Supplementary Fig. S16D, E). These results highlight Zbtb7b as a potential target molecule for combination therapy to enhance the efficacy of PD-L1 monoclonal antibody therapy.

### CM10 and PD-L1 mAb treatment synergizes in a mouse model

**Fig. 6:**
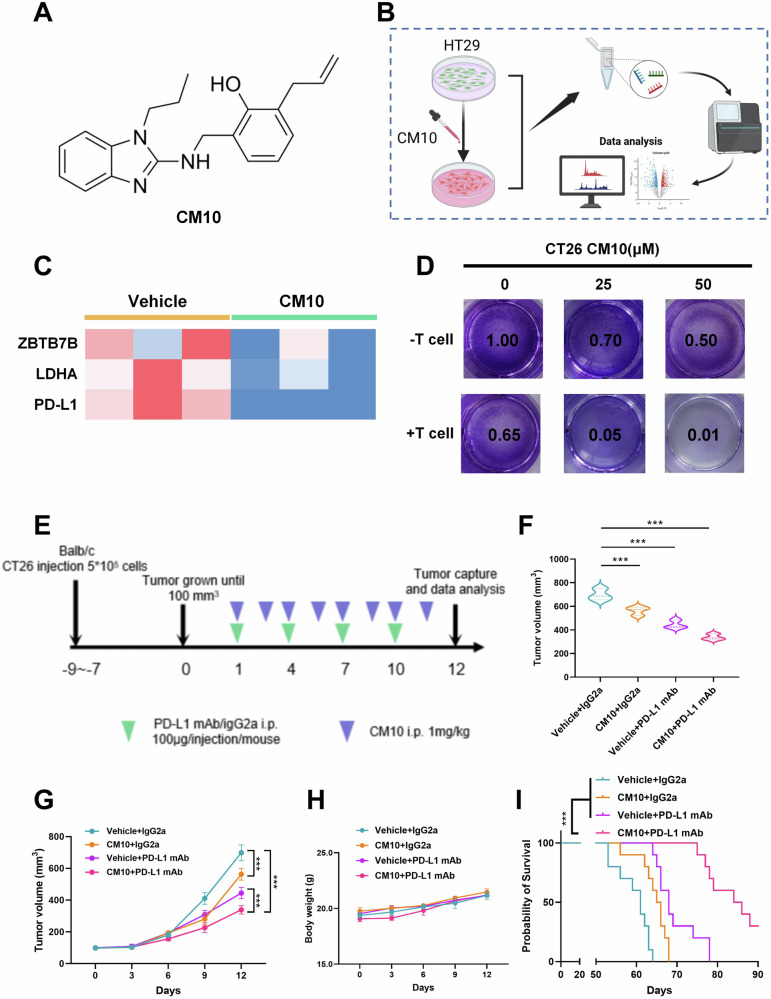
CM10 was able to synergize with PD-L1 mAb therapy to treat tumors in vivo. **A** Chemical structure diagram of CM10. **B** Schematic diagram of the construction of RNA-sequencing in CM10 (10 μM)-treated HT29 human colon cancer cell line and CTRL cells for 2 h. **C** RNA-seq showed the heat map of ZBTB7B, LDHA, and PD-L1 expression between CM10 (10 μM) treated cells and control cells. **D** T cell killing assay of CT26 cells under different concentrations of CM10 (0, 25, 50 μM). **E**–**I** BALB/c mice were implanted with 5 × 10^5^ CT26 cells and received CM10 or PD-1 mAb treatment. **E** Scheme schematic of a treatment timeline. **F** Volume statistics of CT26 tumors harvested after euthanasia of BALB/c xenografted mice. **G** Line graph of tumor volume over time. **H** Mouse body weight was measured every 3 days. **I** Kaplan–Meier survival curves for each group. Results were presented as mean ± S.D., *n* = 3–5. ns not significant; *P < 0.05, **P < 0.01, ***P < 0.001.

Prior experimental results have demonstrated that Aldh1a1, as a crucial upstream molecule of Zbtb7b, contributes to the immune evasion of cancer cells. Subsequently, we investigated whether Aldh1a1 could serve as a potential dual therapeutic target with PD-L1 mAb. CM10, a potent and selective inhibitor of ALDH1A (Fig. 6A), known for its tumor-suppressive effects [34], was employed. We constructed CM10-treated HT29 cells and conducted RNA-sequencing analysis with control cells (Fig. 6B). CM10 could effectively inhibit the enzymatic activity of ALDH (Supplementary Fig. S17). RNA-seq verification revealed that CM10 could reduce the expression of ZBTB7B, LDHA, and PD-L1 (Fig. 6C). In addition, western blot analysis found that CM10 can inhibit ZBTB7B, LDHA, and PD-L1 protein expression in different cells (HT29, CT26, and LLC) (Supplementary Fig. S18). This indicated that CM10 can inhibit its downstream, including ZBTB7B, LDHA and PD-L1, by reducing the activity of ALDH1A1. T cell-mediated cancer cell killing experiments showed that CM10 enhanced the killing ability of T cells in CT26 and LLC cells, with increasing doses of CM10 further augmenting the killing ability (Fig. 6D and Supplementary Fig. S19).

To assess whether CM10 had synergistic antitumor efficacy with PD-L1 mAb, CT26 and LLC cells were separately inoculated into immunocompetent mice and treated with CM10 in combination with PD-L1 mAb or IgG isotype control (IgG2a) (Fig. 6E and Supplementary Fig. S20A). In mice inoculated with CT26 cells, both CM10 and PD-L1 mAb treatments alone significantly reduced the tumor burden. Notably, when CM10 and PD-L1 mAb were combined to treat the mouse tumor model, the tumor burden was minimal (Fig. 6F, G). Similar trends were observed in LLC cells (Supplementary Fig. S20B, C). Moreover, the combination of CM10 and PD-L1 mAb did not cause significant damage to the body weight of mice but extended the survival time of tumor-bearing mice (Fig. 6H, I and Supplementary Fig. S20D, E). These findings suggest that Aldh1a1 could be a potential target for combination therapy with PD-L1 mAb.

### ALDH1A1 and ZBTB7B are associated with the efficacy of PD-1 antibody therapy in NSCLC patients

**Fig. 7:**
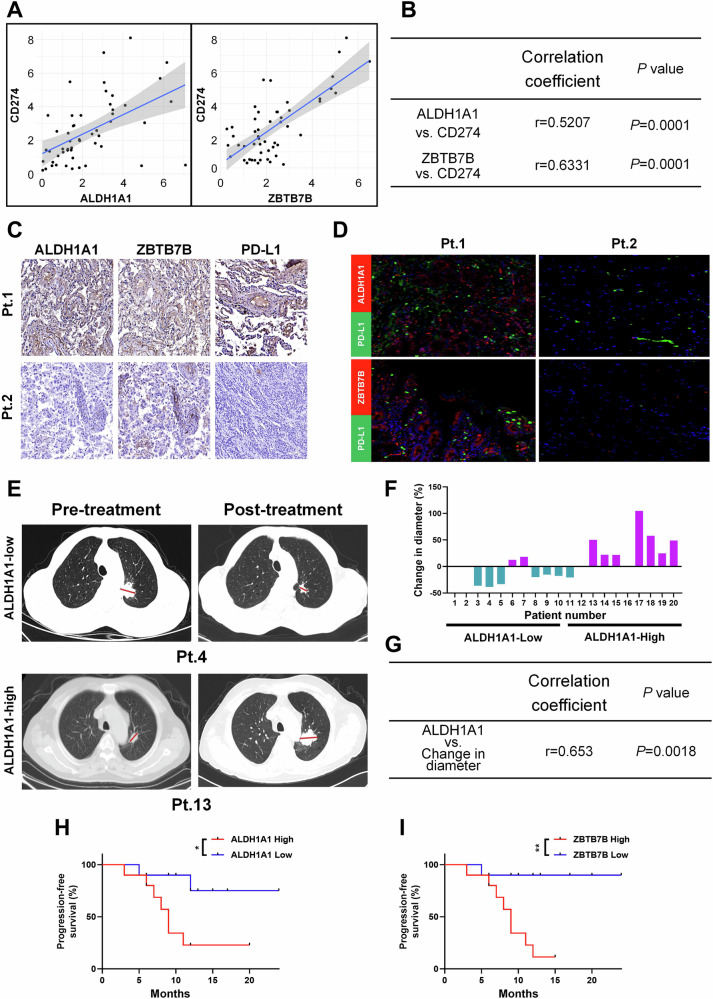
ALDH1A1 and ZBTB7B were positively correlated with PD-L1 expression, respectively, in NSCLC samples. **A**, **B** The correlation of ALDH1A1, ZBTB7B and CD274 expression detected by qPCR in clinical NSCLC tissues. **A** Scatterplot of the correlation of ALDH1A1 and CD274 expression and the correlation of ZBTB7B and CD274 expression. **B** Quantitative correlation between the expression levels of ALDH1A1, ZBTB7B, and CD274. Correlation coefficients r and *p* values were calculated based on Spearman’s rank correlation method. **C** Representative images of immunohistochemical staining for ALDH1A1, ZBTB7B, or PD-L1 expression in NSCLC patient tissues. **D** IF labeling using ALDH1A1, ZBTB7B, and PD-L1 antibodies in tumor tissues from two patients. **E** The radiologist annotated the tumor diameter based on CT imaging with a red line. **F** The difference in tumor diameter before and after treatment with PD-1 monoclonal antibody (Nivolumab) in all patients, where the patients with increased tumor diameter are indicated in purple, and those with decreased tumor diameter are indicated in blue. **G** Quantitative correlation between tumor diameter changes and ALDH1A1 expression levels. Correlation coefficients r and *p* values were calculated based on Spearman’s rank correlation method. **H**, **I** Kaplan–Meier survival curves of PFS in groups with high and low expression of ALDH1A1 (**H**) and ZBTB7B in NSCLC patients. *P < 0.05, **P < 0.01, ***P < 0.001.

To further validate our findings in human cancer patient samples, we collected 52 fresh NSCLC specimens and 32 fresh colon cancer specimens. The mRNA expression levels of ALDH1A1, ZBTB7B, and CD274 were detected. Statistical analysis revealed that in non-small cell lung cancer (NSCLC) and colon cancer, ALDH1A1 and ZBTB7B were positively correlated with CD274 mRNA expression (Fig. 7A, B and Supplementary Fig. S21A, B). Furthermore, we recruited 20 NSCLC patients who received PD-1 mAb therapy (nivolumab) (Supplementary Table S3). The protein expression of ALDH1A1, ZBTB7B, and PD-L1 was evaluated in tumor biopsy tissue. Immunohistochemical quantitative analysis and immunofluorescence results were consistent with correlation analysis, and high expression of ALDH1A1 and ZBTB7B was correlated with high level of PD-L1 (Fig. 7C, D and Supplementary Fig. S22).

Imaging results indicated that cases with low ALDH1A1 expression responded better to PD-1 mAb therapy. Two representative cases of tumor diameter annotated by radiologists are shown in Fig. 7E. Patient 4, with relatively low ALDH1A1 expression, experienced tumor shrinkage after treatment (Fig. 7E, top panel). Conversely, patient 13, with relatively high ALDH1A1 expression, showed tumor growth after treatment (Fig. 7E, bottom panel). The treatment responsiveness of all patients was summarized (Supplementary Table S4 & Fig. 7F), revealing a significant positive correlation between the relative increase in tumor diameter and ALDH1A1 expression level (Fig. 7G).

Subsequently, we evaluated the correlation between ALDH1A1 and ZBTB7B and the prognosis of NSCLC patients. We used immunohistochemical quantitative analysis to divide the samples into ALDH1A1 high expression group and ZBTB7B high and low expression group. As shown in Fig. 7H, I, high expression of ALDH1A1 or ZBTB7B in NSCLC patients treated with PD-1 blockade was associated with shortened progression-free survival (PFS). This suggests that ALDH1A1 and ZBTB7B could potentially serve as predictors of treatment efficacy for PD-1 monoclonal antibodies.

These preclinical results provide further validation of the potential benefits of targeting the ALDH1A1-ZBTB7B axis in tumor patients with high expression of ALDH1A1 and ZBTB7B. Targeted inhibition not only suppresses tumor proliferation but also enhances the efficacy of PD-1 monoclonal antibody therapy by preventing tumor immune escape. While an ALDH1A1 antagonist is not yet FDA-approved for tumor treatment, its potential as a significant anti-tumor drug, synergistically enhancing immunotherapy efficacy, makes it a promising avenue for future research.

## Discussion

The accumulation of lactic acid in the microenvironment, a key influencing factor significantly affecting the tumor immune escape phenotype, has garnered attention. Previous studies have demonstrated that lactate directly inhibits inflammatory signaling pathways and plays a crucial role in regulating macrophage polarization, tumor immunity, and antiviral responses [35]. In addition, the accumulation of lactate in the tissue microenvironment could mediate histone lysine lactylation and regulate the inhibitory effect of various immune cells [36, 37]. In this study, we found that ALDH1A1 induces lactate accumulation by regulating the transcription of LDHA through direct transcriptional regulation of the downstream ZBTB7B. Notably, ALDH1A1 has been abundantly expressed in early colon cancer [38], and in breast cancer, it reduces the pH value in tumor cells, activating the inflammatory signal NF-κB, resulting in myeloid cell suppression and immune suppression [16]. Our functional assays using a T cell-mediated cancer cell killing assay demonstrated that ALDH1A1 deficiency in various tumor cells significantly enhances T cell-mediated cytotoxicity by suppressing tumor glycolytic levels. Additionally, our investigation into the post-translational modification phenotype of ZBTB7B revealed its SUMO2ylation at specific sites, which, unlike ubiquitination, stabilizes the modified protein, particularly transcription factors, significantly enhancing their function [39, 40].

Although immune checkpoint blockade with PD-1 or PD-L1 mAbs provides substantial benefits to patients with advanced cancers, the overall response rate is low, often accompanied by drug resistance. Our preclinical animal studies demonstrated that inhibiting the ALDH1A1-ZBTB7B regulatory axis synergized with PD-1 mAb treatment, leading to a significant reduction in tumor volume and prolonged survival compared to the control group. This finding highlights the potential of combination therapeutic strategies for tumor immunotherapy.

In NSCLC samples, we observed a positive correlation between ALDH1A1-ZBTB7B and PD-L1 expression. Evaluation of the clinical significance of the ALDH1A1-ZBTB7B axis in NSCLC patients treated with PD-1 mAb revealed that high expression indicated poorer therapeutic outcomes in NSCLC patients responding to PD-1 mAb therapy. This suggests potential screening strategies for assessing the therapeutic effect of PD-1 mAbs.

We inhibited ALDH1A1 by knockdown and CM10 respectively. After inhibiting ALDH1A1 enzyme activity by the small molecule inhibitor CM10, it can produce a phenotype similar to inhibiting ALDH1A1 expression, which indicates that the catalytic activity of ALDH1A1 plays a key role in its biological function. However, there are differences in the specific effects of these two modes of inhibition within cells. Inhibiting the expression of ALDH1A1 enzyme may affect all reactions that depend on this enzyme, while inhibiting enzyme activity may only affect specific metabolic pathways. In addition, long-term inhibition of enzyme expression in vivo may lead to changes in cellular adaptability, such as the generation of compensatory mechanisms, whereas inhibition of enzyme activity may not cause such adaptive changes. Therefore, we believe that it is a safer strategy to elucidate the mechanism and then develop suitable inhibitors.

In summary, our study underscores the crucial role of glycolysis in tumor cells in influencing immune evasion. ZBTB7B, a transcription factor of the rate-limiting enzyme LDHA involved in lactate production in glycolysis, emerges as a critical gene contributing to the failure of tumor cell immunotherapy. The combination of ALDH1A1 inhibitors and PD-1 mAb represents a promising therapeutic strategy to mitigate tumor immune escape. Consequently, the development of FDA-approved ALDH1A1 antagonists for use in combination with current anti-PD-1/PD-L1 therapies warrants attention.

## Conclusions

Our study has elucidated the pivotal role of glycolysis activation in tumor cells in facilitating immune evasion. Additionally, we identified that ALDH1A1 exacerbates the immune evasion characteristics of tumor cells by modulating the ZBTB7B-glycolysis regulatory pathway. The co-administration of PD-1 mAb and an ALDH1A1 antagonist enhances the anti-tumor therapeutic effect by augmenting the activity of killer CD8+ T cells. This research unveils the molecular mechanisms governing the tumor immune escape phenotype and sheds light on potential combination therapeutic strategies for immunotherapy.

### Supplementary information

Supplementary material

Uncropped WB images

## Data Availability

All data needed to evaluate the conclusions are present in the paper. The RNA-seq data were deposited to the GEO database with the accession number GSE225822 and GSE269935. Other data and materials are available upon reasonable request.
