# Supplementary material for: ALDH1A1 promotes immune escape of tumor cells through ZBTB7B-glycolysis pathway
Source: Cell Death Dis. 2024 Aug 7;15(8):568. doi: 10.1038/s41419-024-06943-9 (PMC11303523; doi:10.1038/s41419-024-06943-9)
Supplement: Supplementary file 1 — Supplementary material [file 41419_2024_6943_MOESM1_ESM.docx]

**Supporting Information**

**ALDH1A1 Promotes Immune Escape of Tumor Cells through ZBTB7B-****Glycolysis Pathway**

Mingyuan Wang^1,2,3^, Taoli Wang^4^, Jinjin Wang^5^, Yuexin Yang^6^, Xi Li^1,2,3^, Huan Chen^5^ and Jingnan Liao^7^

**Author details**

1 Department of Geratic Surgery, Xiangya Hospital, Central South University, Changsha, Hunan, China.

2 National Clinical Research Center for Geriatric Disorders, Xiangya Hospital, Central South University, Changsha, Hunan, China.

3 Department of General Surgery, Xiangya Hospital, Central South University, Changsha, Hunan, China.

4 Department of Pathology, the Affiliated Zhuzhou Hospital Xiangya Medical College, Central South University, Zhuzhou, Hunan, China.

5 Department of Gynaecology, the Affiliated Zhuzhou Hospital Xiangya Medical College, Central South University, Zhuzhou, Hunan, China.

6 Department of Oncology, the Affiliated Zhuzhou Hospital Xiangya Medical College, Central South University, Zhuzhou, Hunan, China.

7 Hunan Provincial Key Laboratory of Regional Hereditary Birth Defects Prevention and Control, Changsha Hospital for Maternal & Child Health Care Affiliated to Hunan Normal University, Changsha, China.

**Correspondence**

Jingnan Liao, Hunan Provincial Key Laboratory of Regional Hereditary Birth Defects Prevention and Control, Changsha Hospital for Maternal & Child Health Care Affiliated to Hunan Normal University, Changsha, China.

Email: liaojingnanalice@outlook.com

Huan Chen, Department of Gynaecology, the Affiliated Zhuzhou Hospital Xiangya Medical College, Central South University, Zhuzhou, Hunan, China.

Email: chenhuancsu@163.com

Xi Li, Department of Geratic Surgery, Department of Geratic Surgery, Department of General Surgery, Xiangya Hospital, Central South University, Changsha, Hunan, China.

Email: xilixyyy@163.com

**
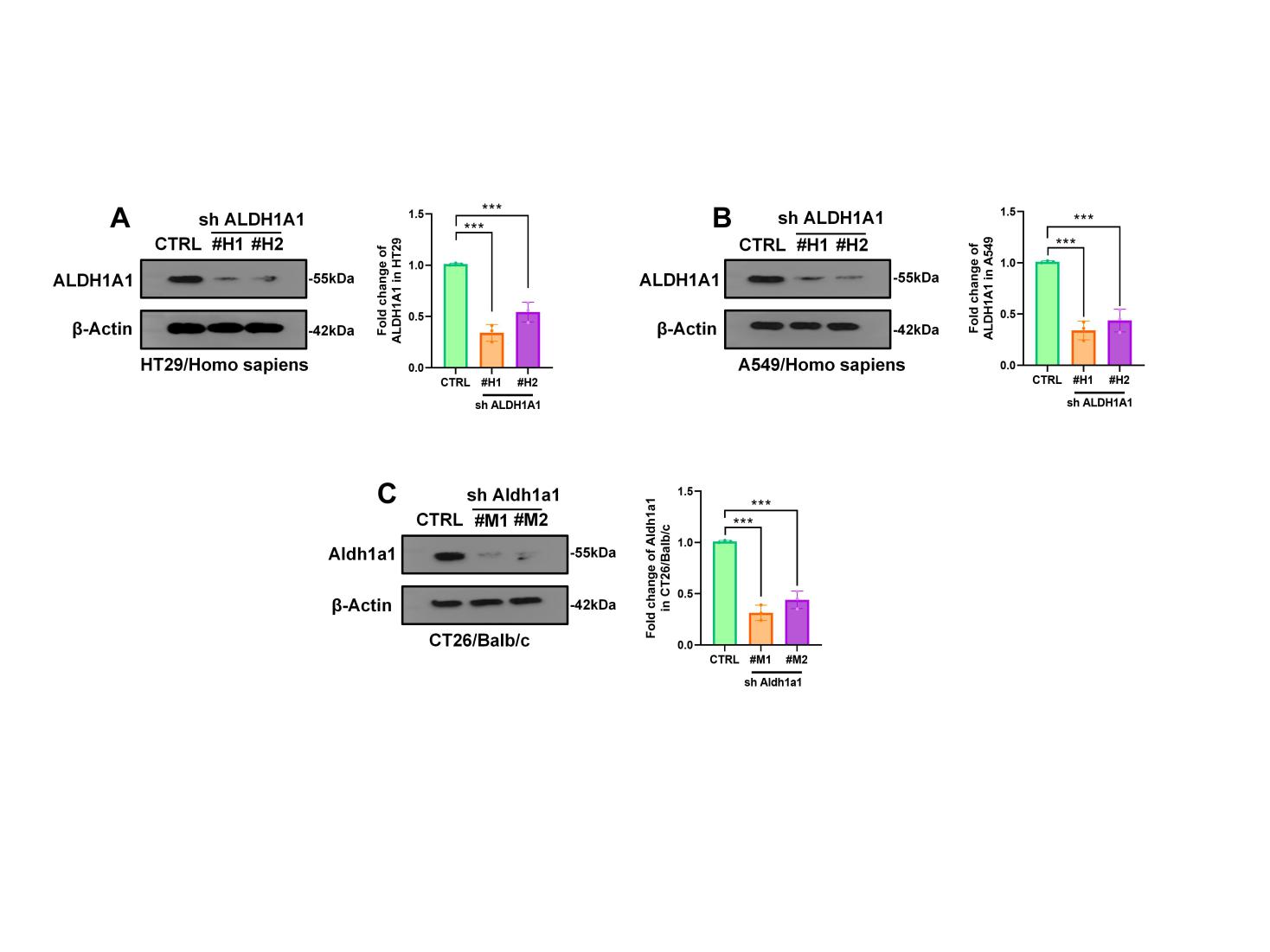
**

**Figure S1** (A-B) Western blot and histogram of the effect of shALDH1A1 (#H1 and #H2) knocking down ALDH1A1 in different cells (HT29 and A549), respectively. (C) Western blot and histogram of the effect of shAldh1a1 (#M1 and #M2) knocking down Aldh1a1 in CT26 cells. Results were presented as mean ± S.D., n = 3. **P* < 0.05, ***P* < 0.01, ****P* < 0.001.


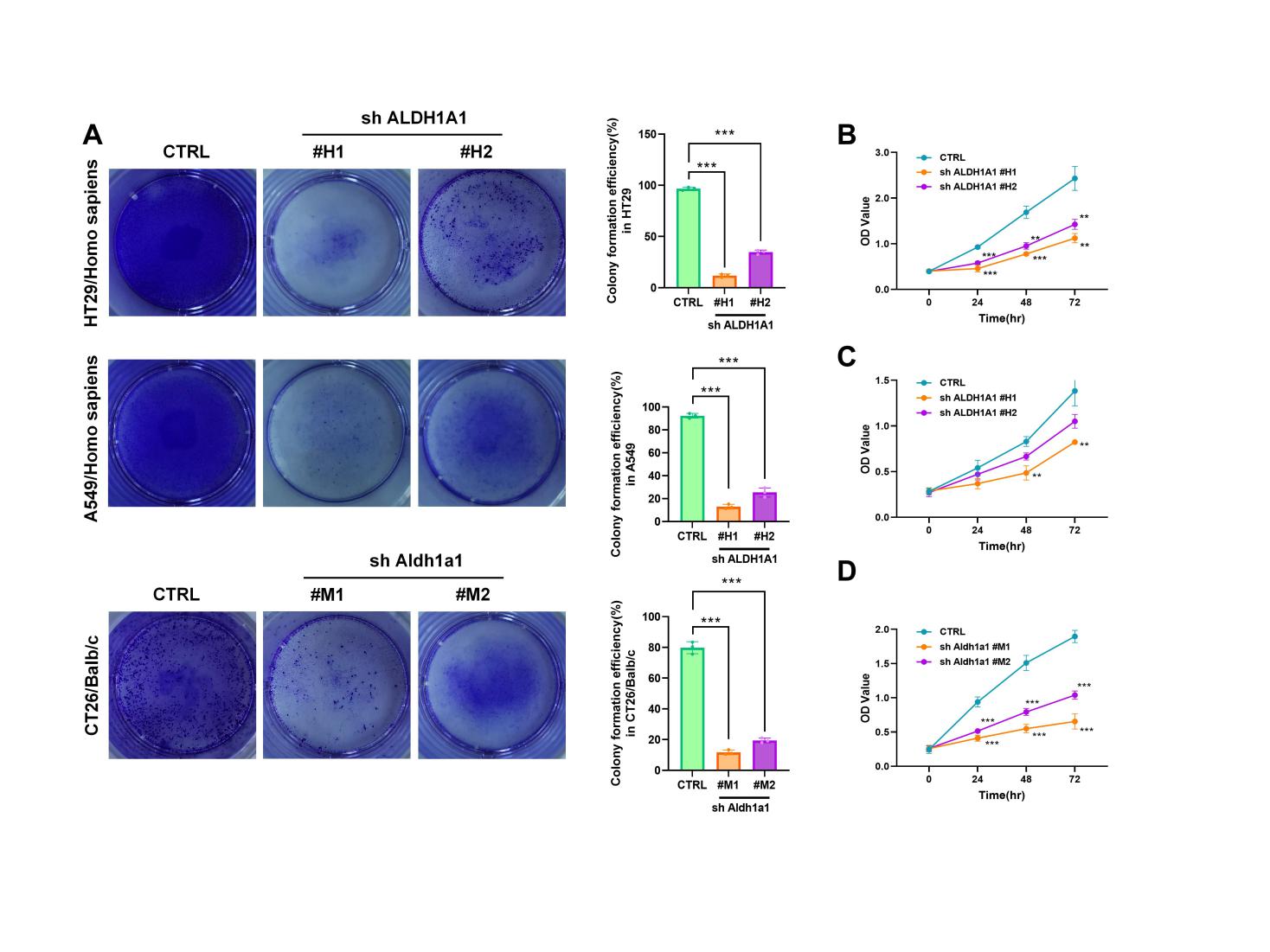


**Figure S2** The effect of shALDH1A1 on cell proliferation in three kinds of cell lines HT29, A549 and CT26. (A) Clonogenic experiments and statistics. (B) Statistics of cell proliferation experiments. Results were presented as mean ± S.D., n = 3. ***P* < 0.01, ****P* < 0.001.

**
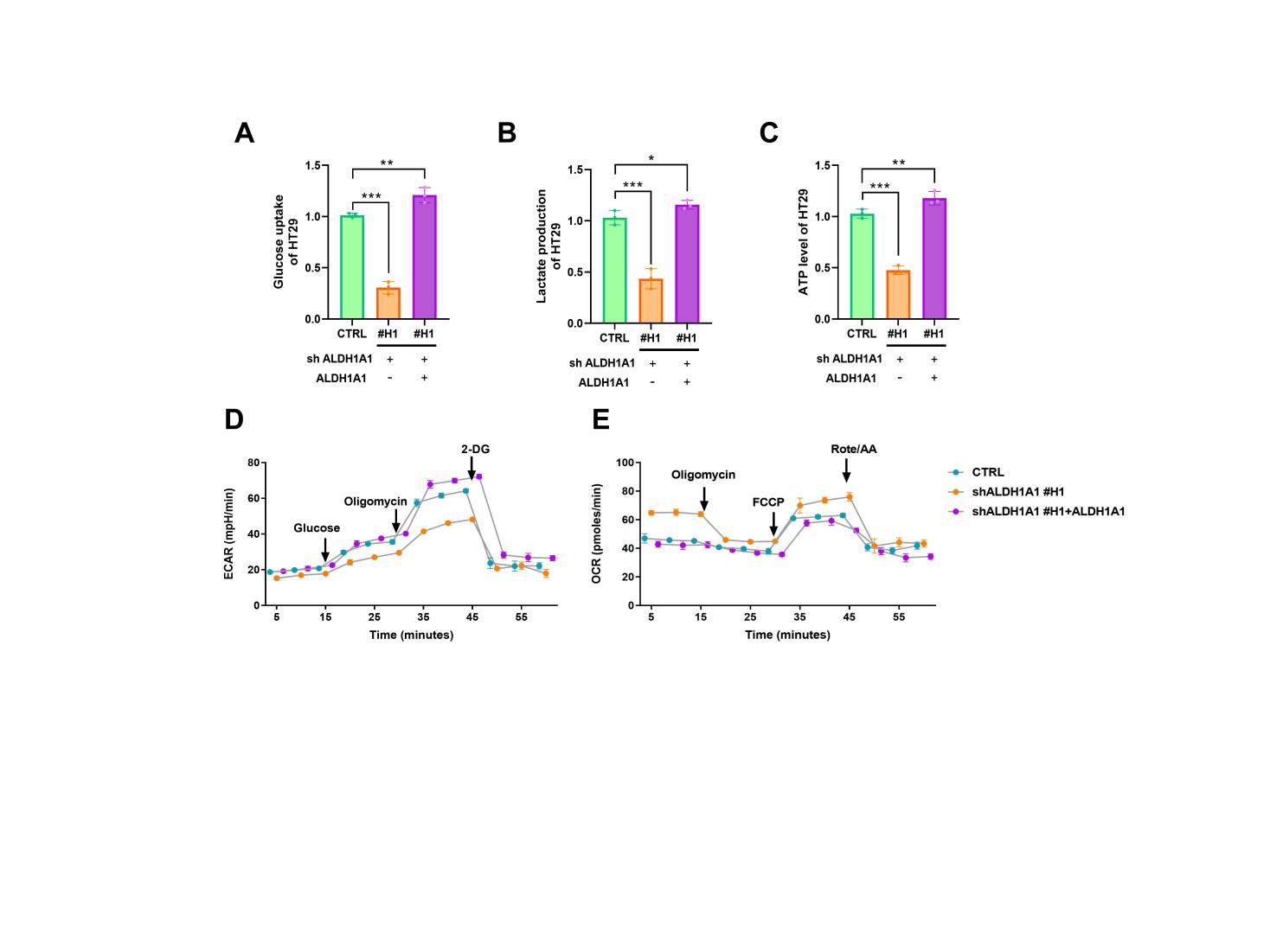
**

**Figure S3** Changes in key indicators of glycolysis in HT29 after shALDH1A1 knockdown and compensation. (A) Glucose uptake. (B) Lactate production levels. (C) ATP generation levels. (D) Extracellular acidification rate (ECAR). (E) Oxygen consumption (OCR).

**
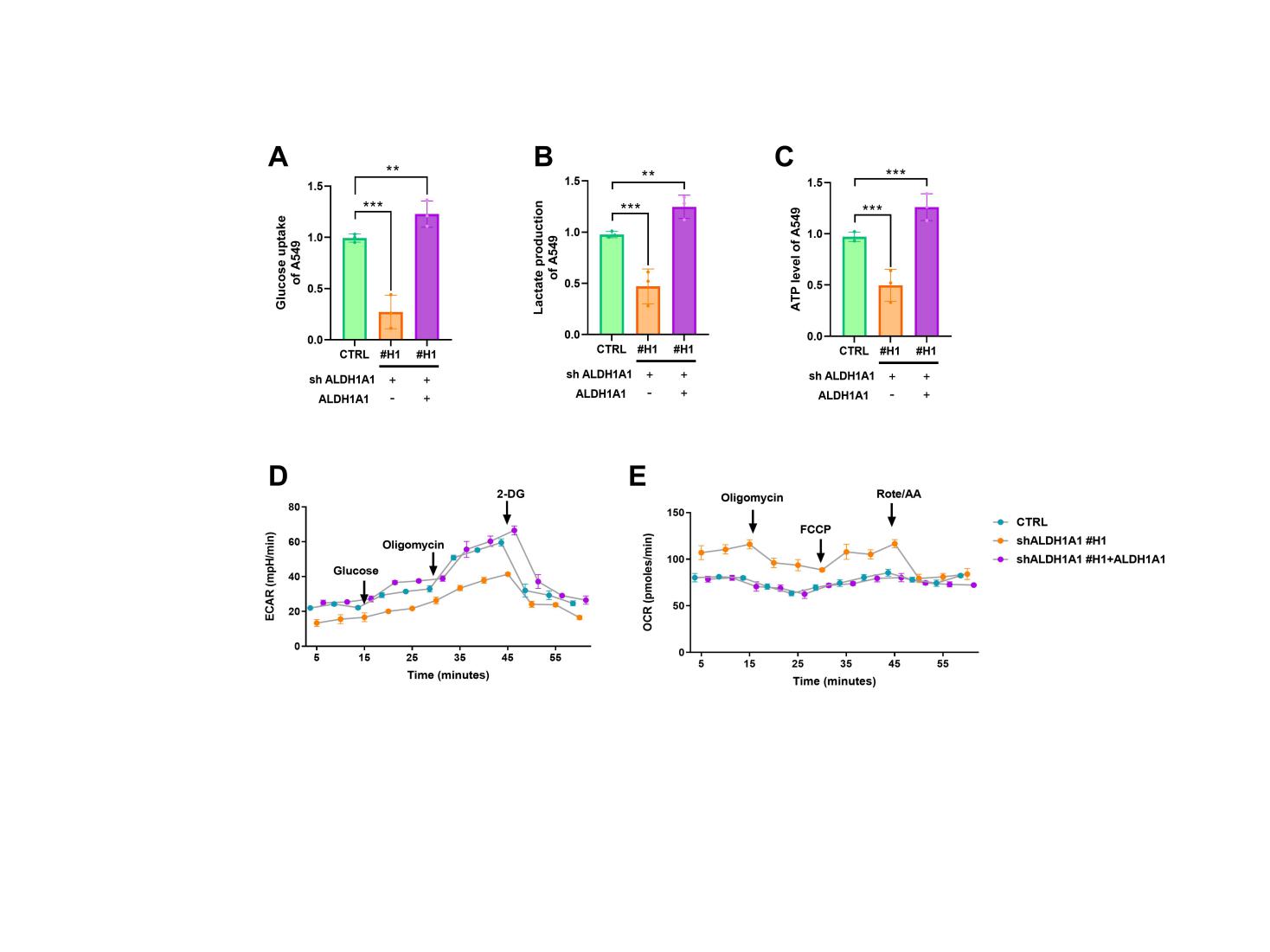
**

**Figure S4** Changes in key indicators of glycolysis in A549 after shALDH1A1 knockdown and compensation. (A) Glucose uptake. (B) Lactate production levels. (C) ATP generation levels. (D) Extracellular acidification rate (ECAR). (E) Oxygen consumption (OCR).

**
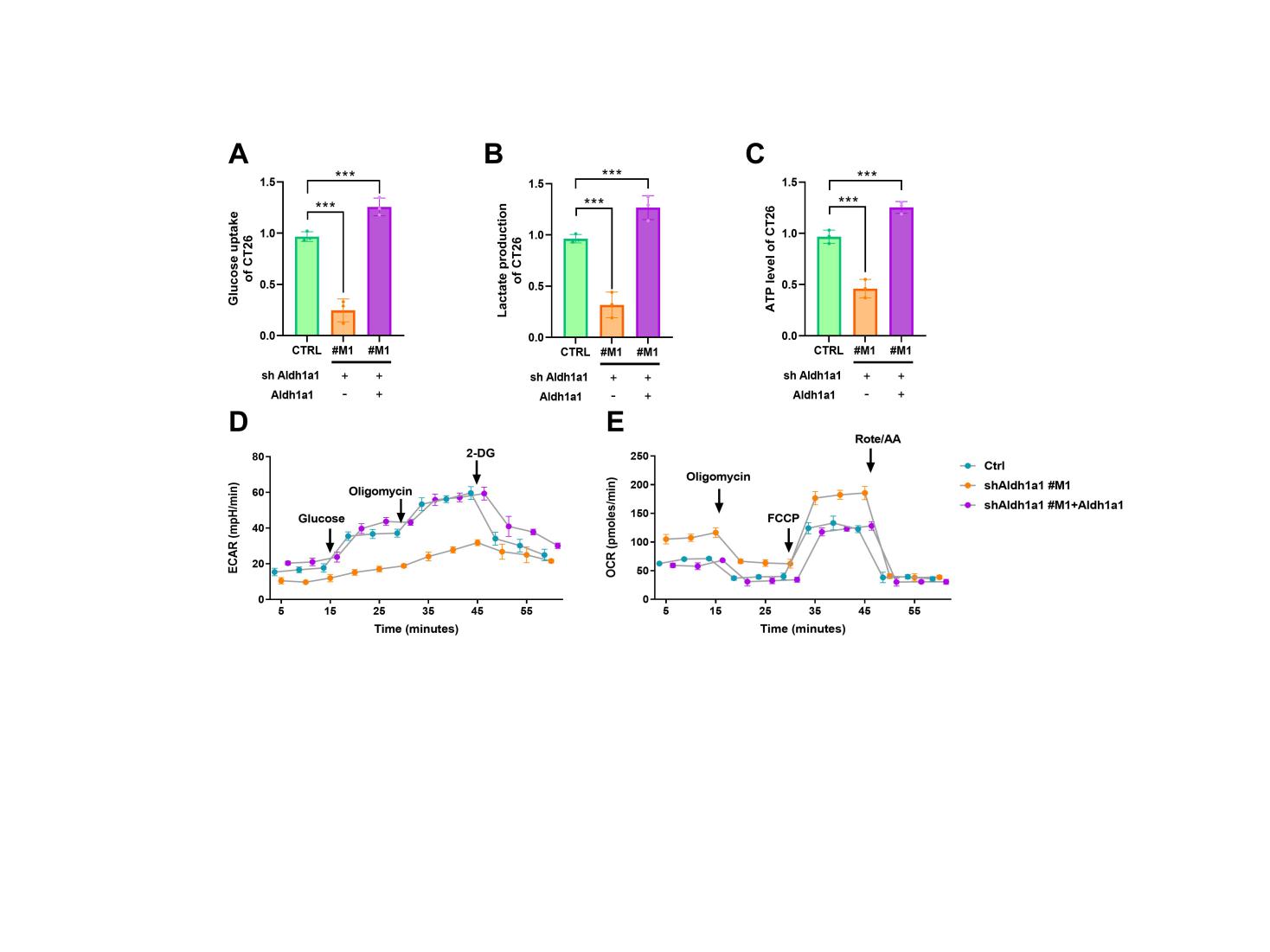
**

**Figure S5** Changes in key indicators of glycolysis in CT26 after shAldh1a1 knockdown and compensation. (A) Glucose uptake. (B) Lactate production levels. (C) ATP generation levels. (D) Extracellular acidification rate (ECAR). (E) Oxygen consumption (OCR).

**
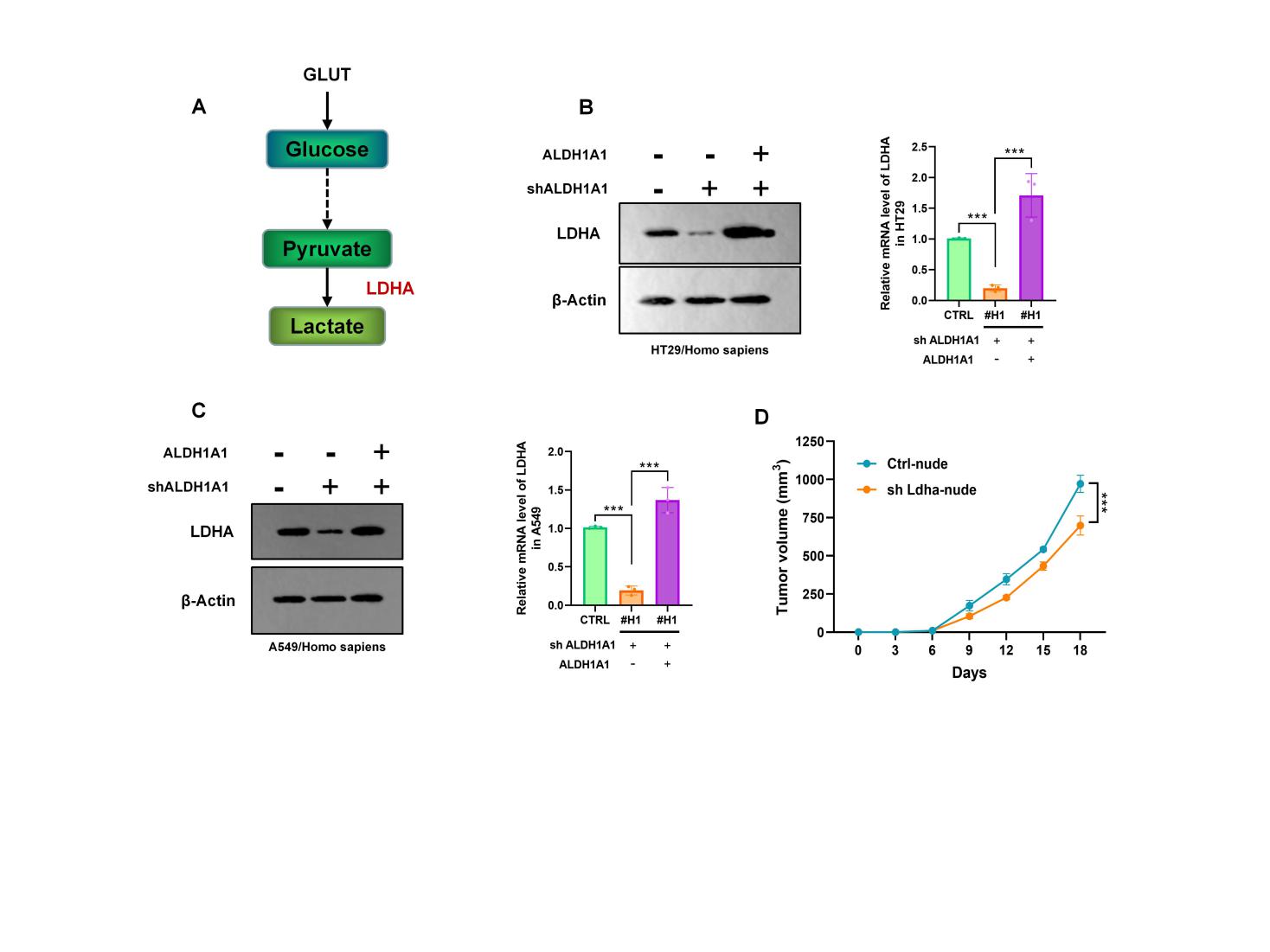
**

**Figure S6** (A) Schematic diagram of the signaling pathway for the mechanism of glycolysis. (B-C) Western blot and qPCR detection of LDHA expression changes in HT29 and A549 cells after shALDH1A1 knockdown and compensation. (D) Tumor growth curves of BALB/c nude mouse species confounding negative control (Ctrl) and shLdha-transfected CT26 xenografts.

**
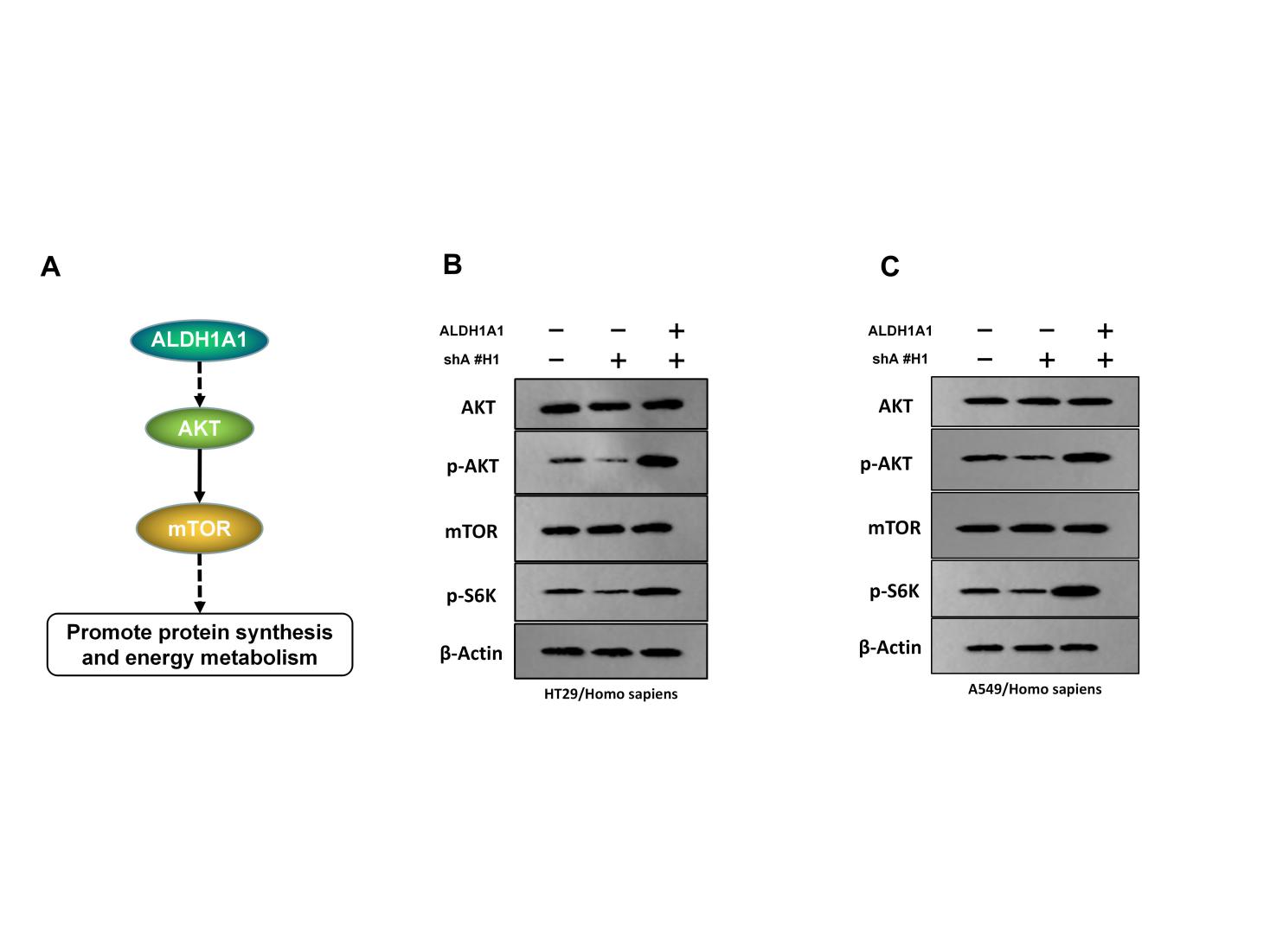
**

**Figure S7** (A) Schematic diagram of the signaling pathway downstream of ALDH1A1 regulating AKT/mTOR. (B-C) The effect of shALDH1A1 knockdown and compensation on the expression of proliferation signal AKT/p-AKT/mTOR/p-S6K in different cells HT29 (B) and A549 (C).

**
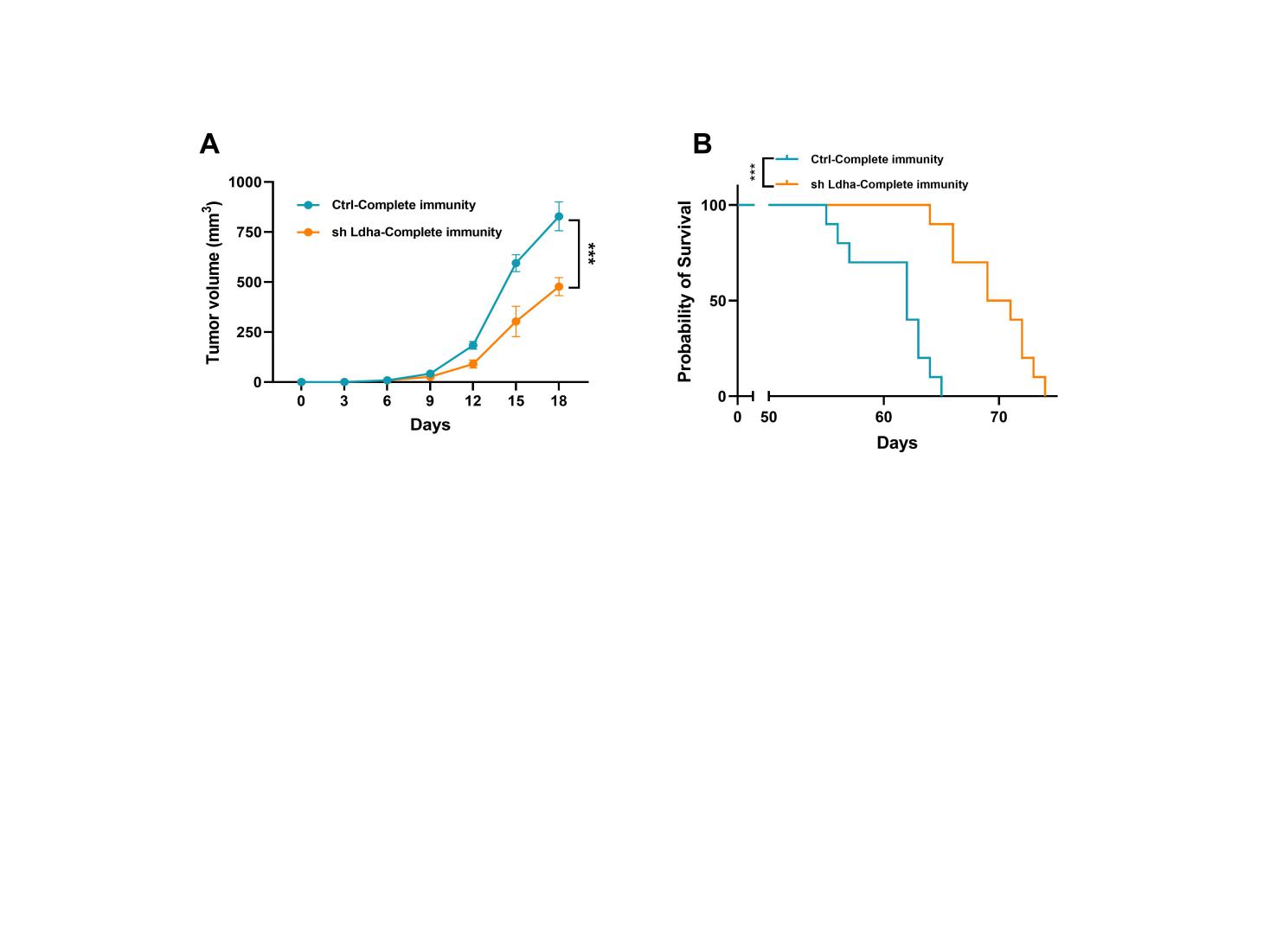
**

**Figure S8** Tumor growth curves (A) of confounding negative control (Ctrl) and shLdha-transfected CT26 xenografts in BALB/c mice and Kaplan-Meier survival curves (B) of these mice.

**
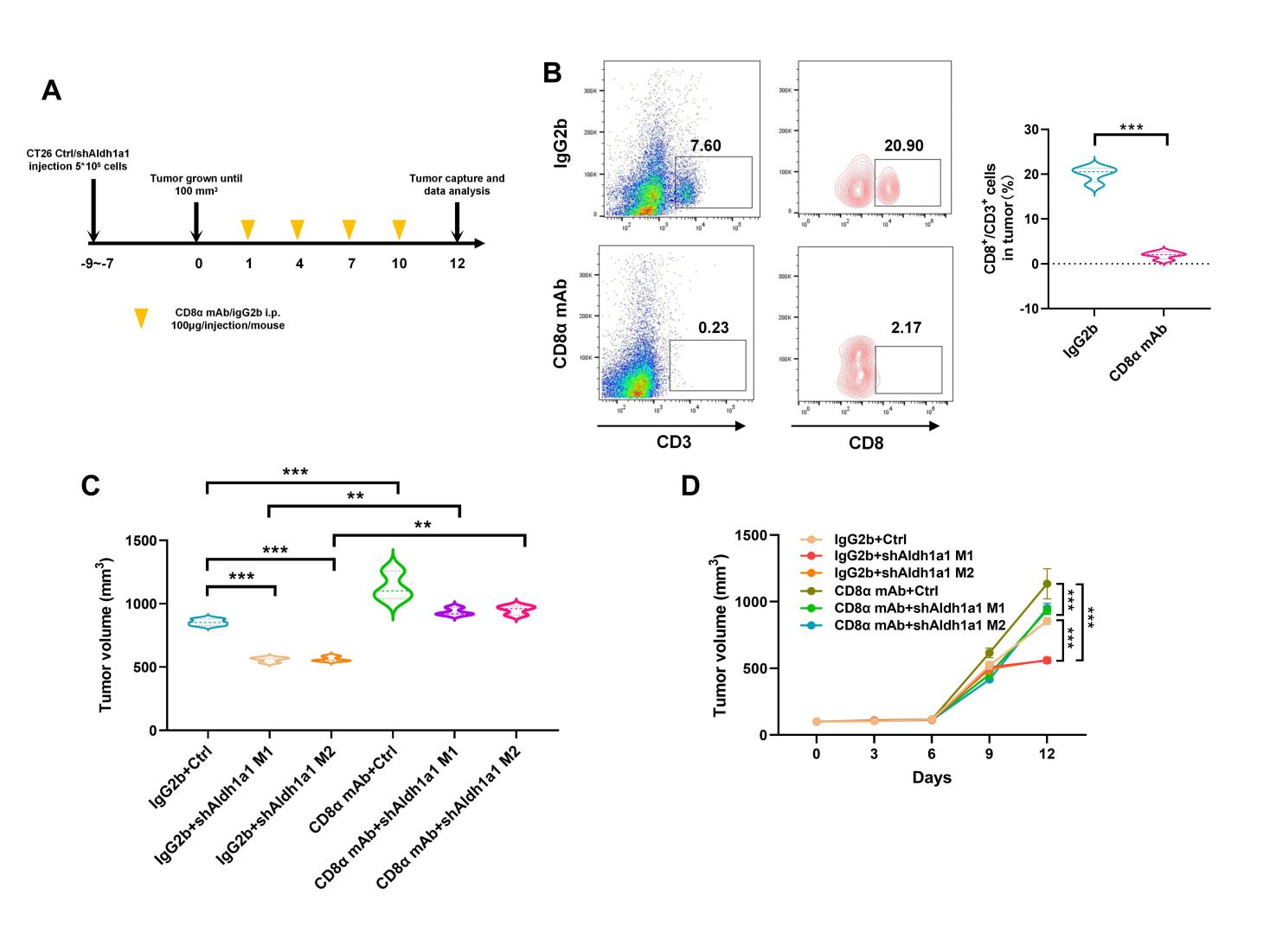
**

**Figure S9** (A–D) BALB/c mice were implanted with 5×10^5^ shAldh1a1 CT26 or Ctrl cells and received CD8α mAb treatment or IgG isotype control (IgG2b). (A) Schematic diagram of the treatment timeline. (B) Flow cytometric sorting of CD3^+^CD8^+^ TILs and quantitative statistics in tumorigenic tissues of BALB/c mice treated with CD8α mAb or IgGb. (C) Volume statistics of CT26 tumors harvested after euthanasia of BALB/c xenografted mice. (D) Line graph of tumor volume over time. Results are presented as mean ± S.D., n = 3-5. ***P* < 0.01, ****P* < 0.001.


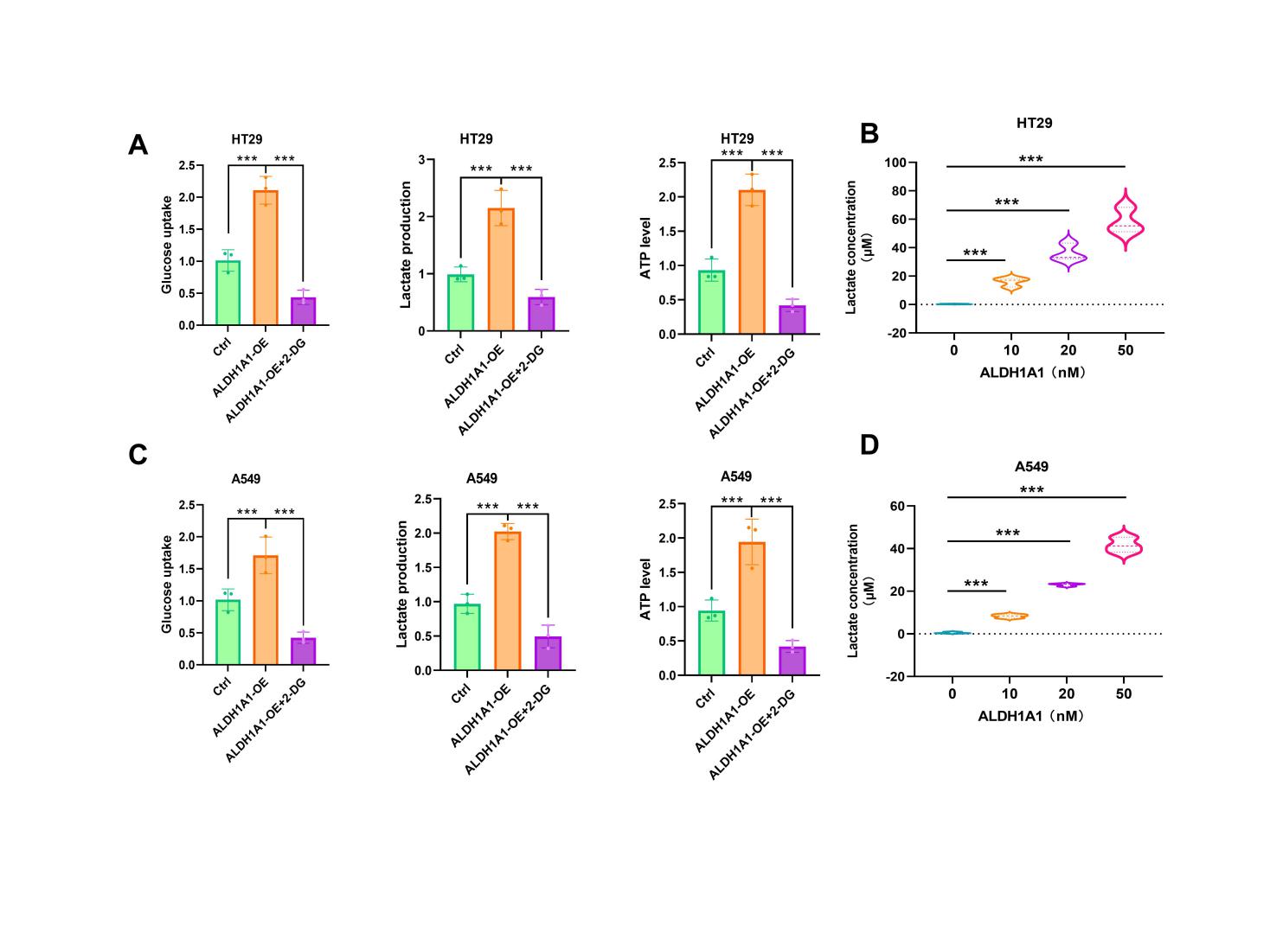


**Figure S10** Histogram of key markers of glycolysis, glucose uptake, lactate production, and ATP levels after ALDH1A1-OE overexpression and 2-DG inhibition of glycolysis in HT29 (A) and A549 (C) cells. After HT29 (B) and A549 (D) cells were treated with ALDH1A1 enzyme at different concentration gradients for 10 minutes, the quantitative statistical graph of lactic acid content in the cell supernatant was detected. Results were presented as mean ± S.D., n = 3. ****P* < 0.001.

**
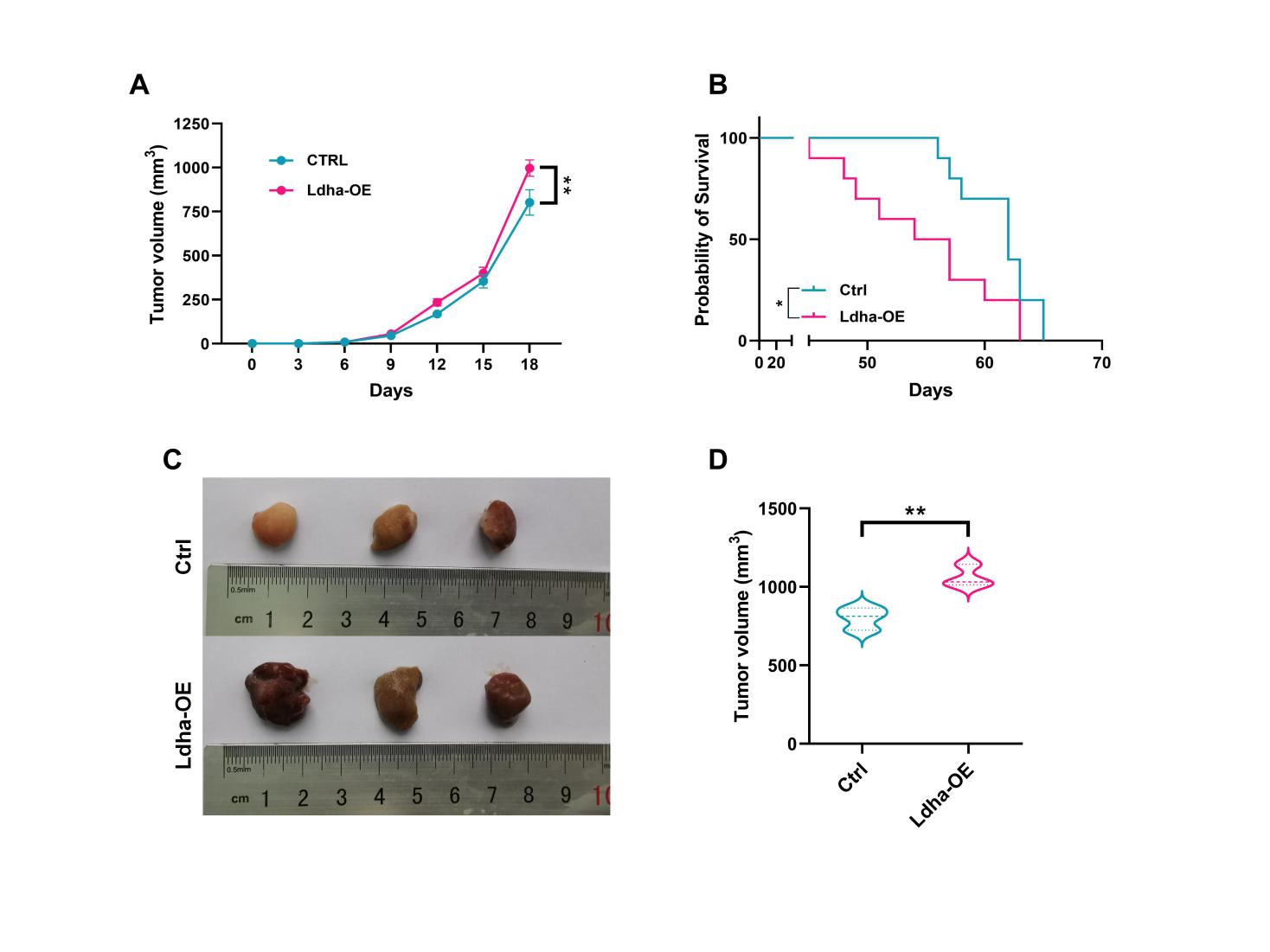
**

**Figure S11** Tumor growth curves (A) of confounding negative control (Ctrl) and Ldha-OE-transfected CT26 xenografts in BALB/c mice and Kaplan-Meier survival curves (B) of these mice. (C-D) BALB/c mice were implanted with CT26 cells and Ldha-overexpressing CT26 cells. Tumors were excised and measured 3 weeks later.

**
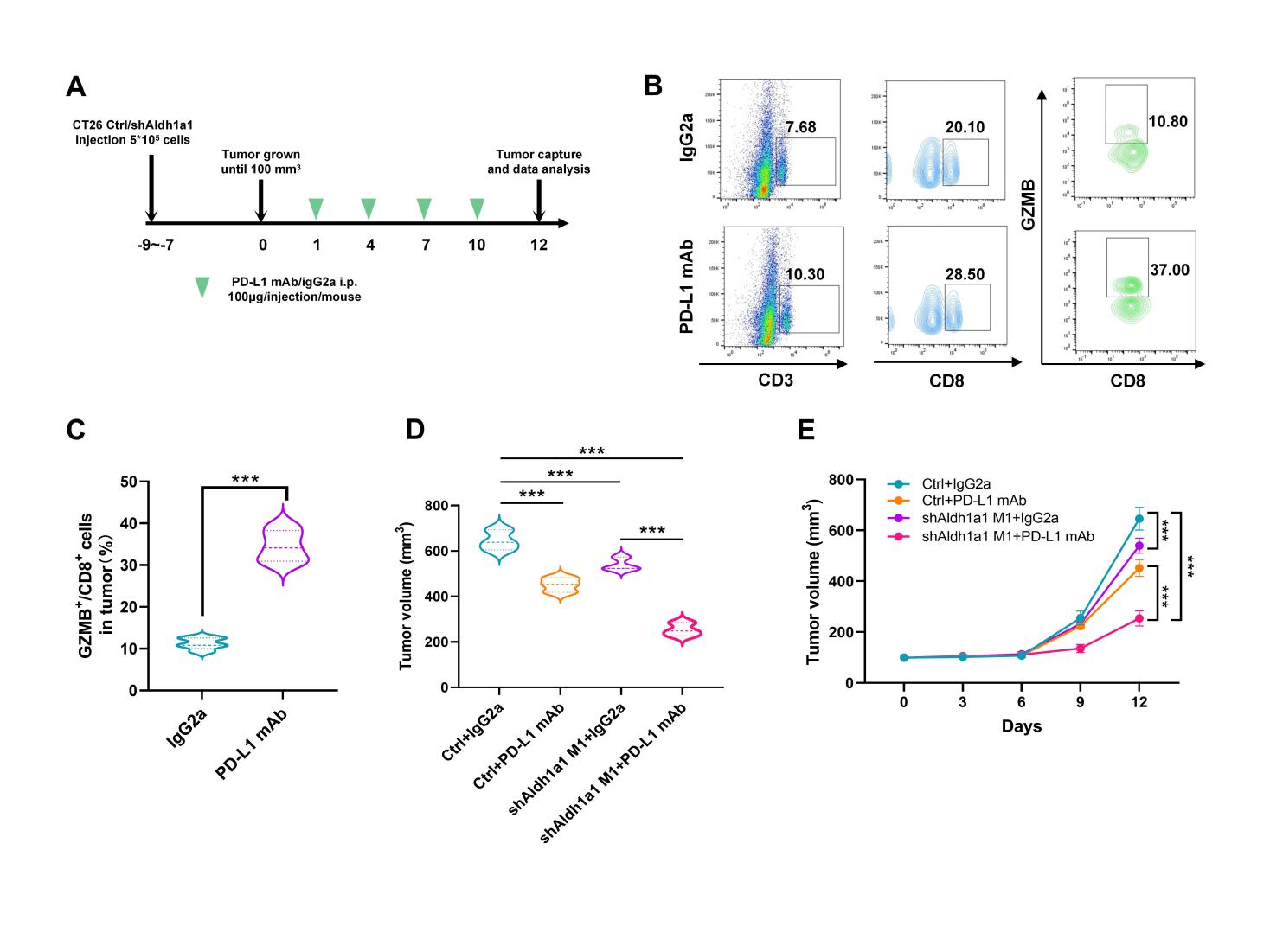
**

**Figure S12** (A-D) BALB/c mice were implanted with 5×10^5^ shAldh1a1 CT26 or Ctrl cells and received PD-L1 mAb treatment or IgG isotype control (IgG2a). (A) Schematic diagram of the treatment timeline. (B-C) Flow cytometry sorting of TILs and quantitative statistics of CD8+GZMB+ in tumorigenic tissues of BALB/c mice treated with PD-L1 mAb or IgGa. (D) Volume statistics of CT26 tumors harvested after euthanasia of BALB/c xenografted mice. (E) Line graph of tumor volume over time. Results were presented as mean ± S.D., n = 3-5. ***P* < 0.01, ****P* < 0.001.

**
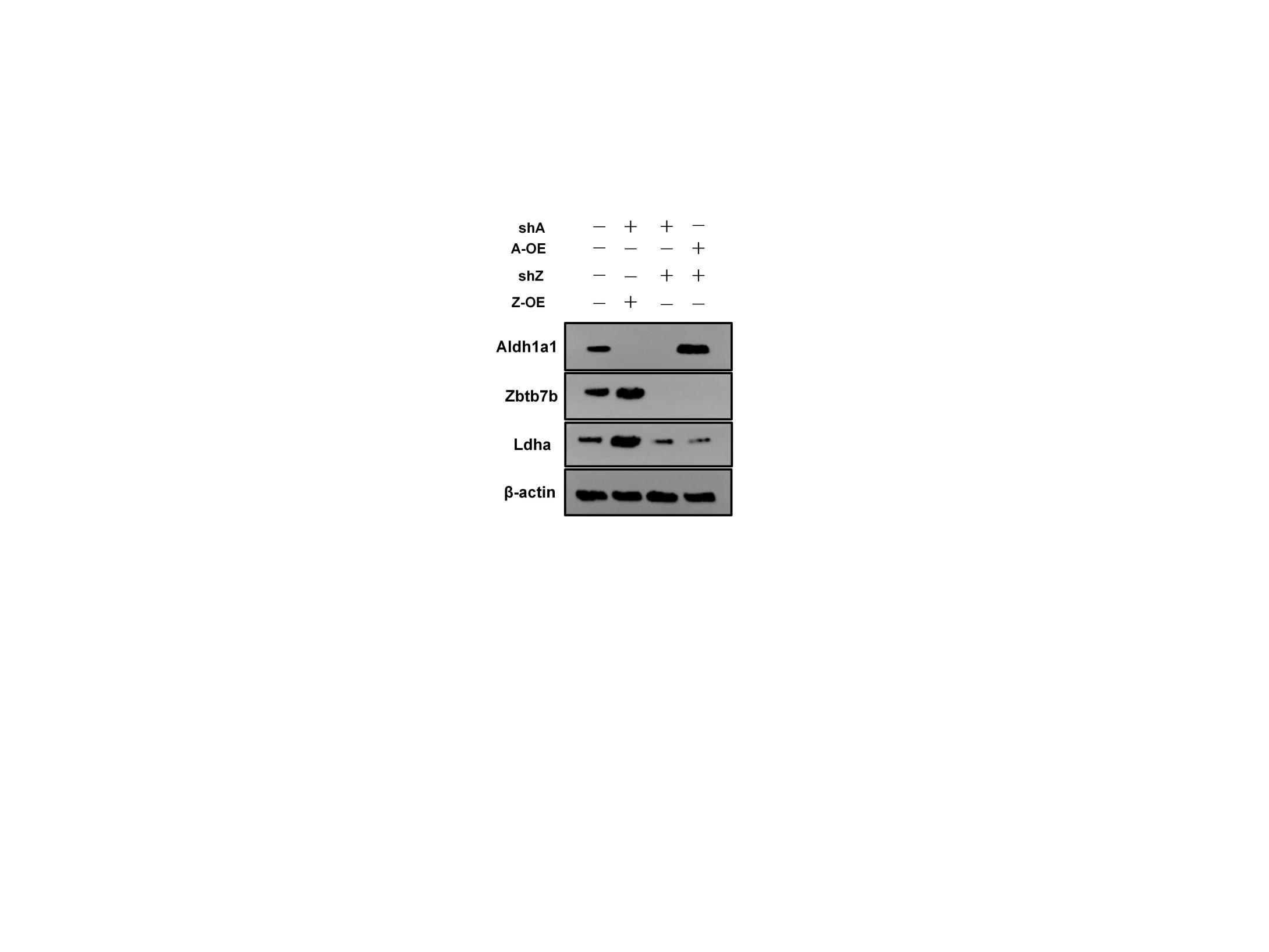
**

**Figure S13** In CT26 cells, Western blot was used to detect the expression changes of Ldha after knockdown (sh-) and overexpression (-OE) of Aldh1a1 and Zbtb7b, respectively.


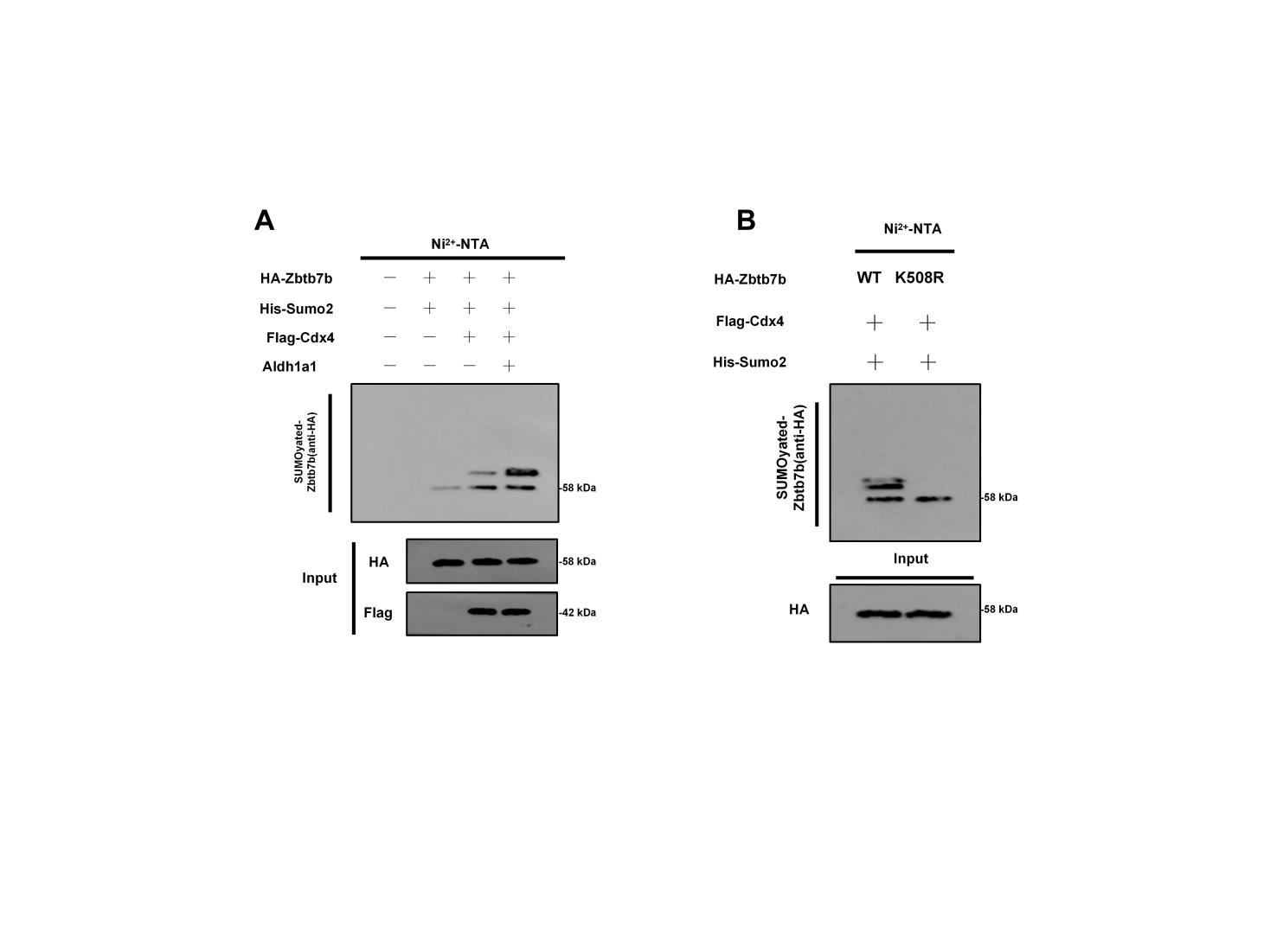


**Figure S14** CT26 cells were transfected with HA-Zbtb7b, Flag-Cdx4, and His-SUMO1/2/3, and the lysate was purified and precipitated by Ni^2+^-NTA agarose bead pull-down method, and finally Western blot was performed with the indicated antibodies. (A) Effect of Aldh1a1 on SUMO2 modification of Zbtb7b in CT26 cells. (B) CT26 cells were transfected with the indicated plasmids HA-Zbtb7b-Wt or Zbtb7b binding site mutant (K508R) and His-SUMO2/Flag-Cdx4, and the lysates were purified and precipitated by Ni2+-NTA agarose bead pull-down method, Finally, Western blot was performed with the designated antibodies. To verify the effect of site mutant K508R on the SUMO2ylation effect of Zbtb7b.

**
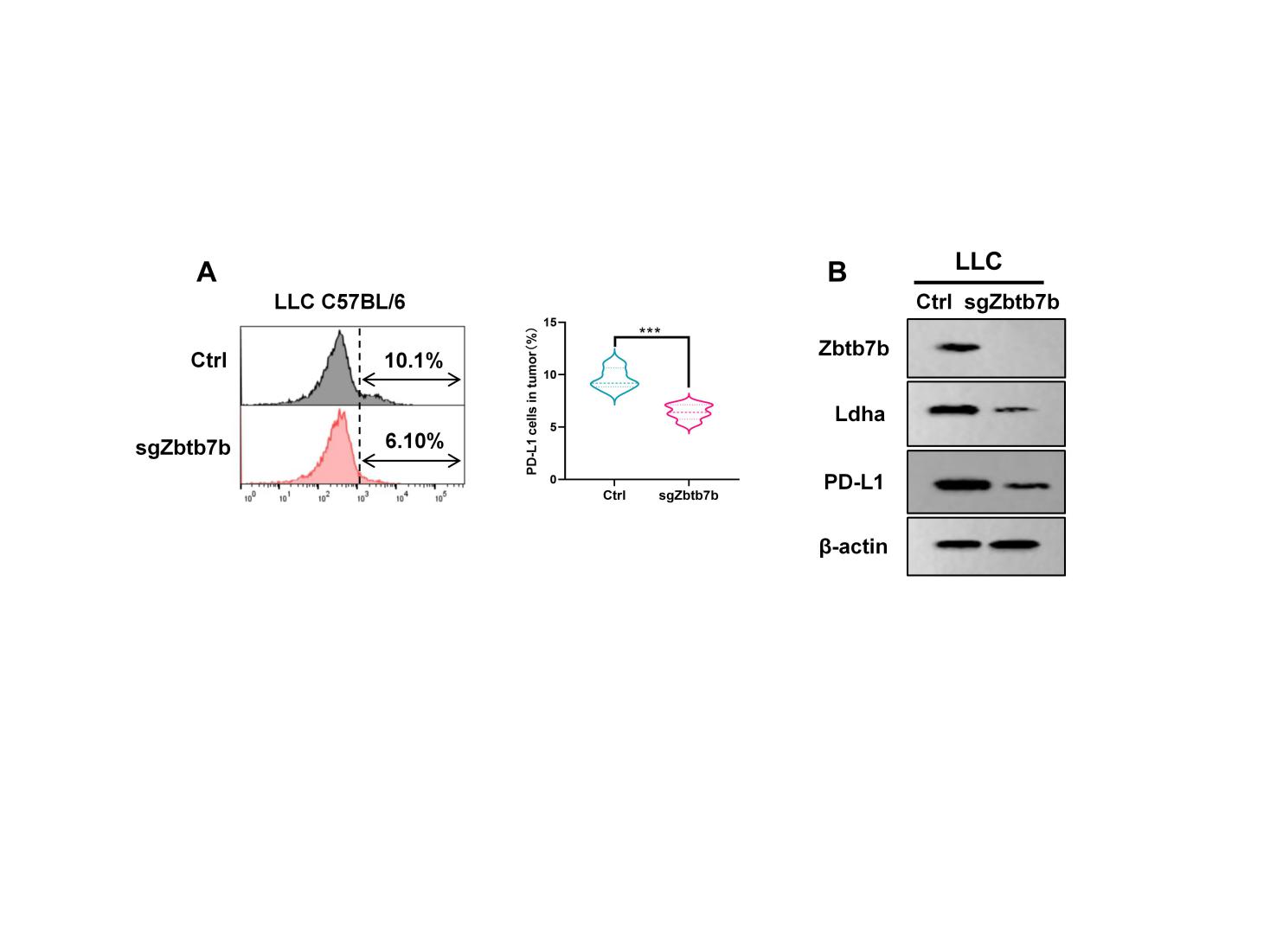
**

**Figure S15** (A) Flow cytometry analysis of PD-L1 expression and quantitative statistics of LLC cell lines transfected with sgZbtb7b or Ctrl sgRNA. (B) Western blot detection of protein expressions of Zbtb7b, Ldha, and PD-L1 in LLC cell lines transfected with sgZbtb7b or Ctrl sgRNA.

**
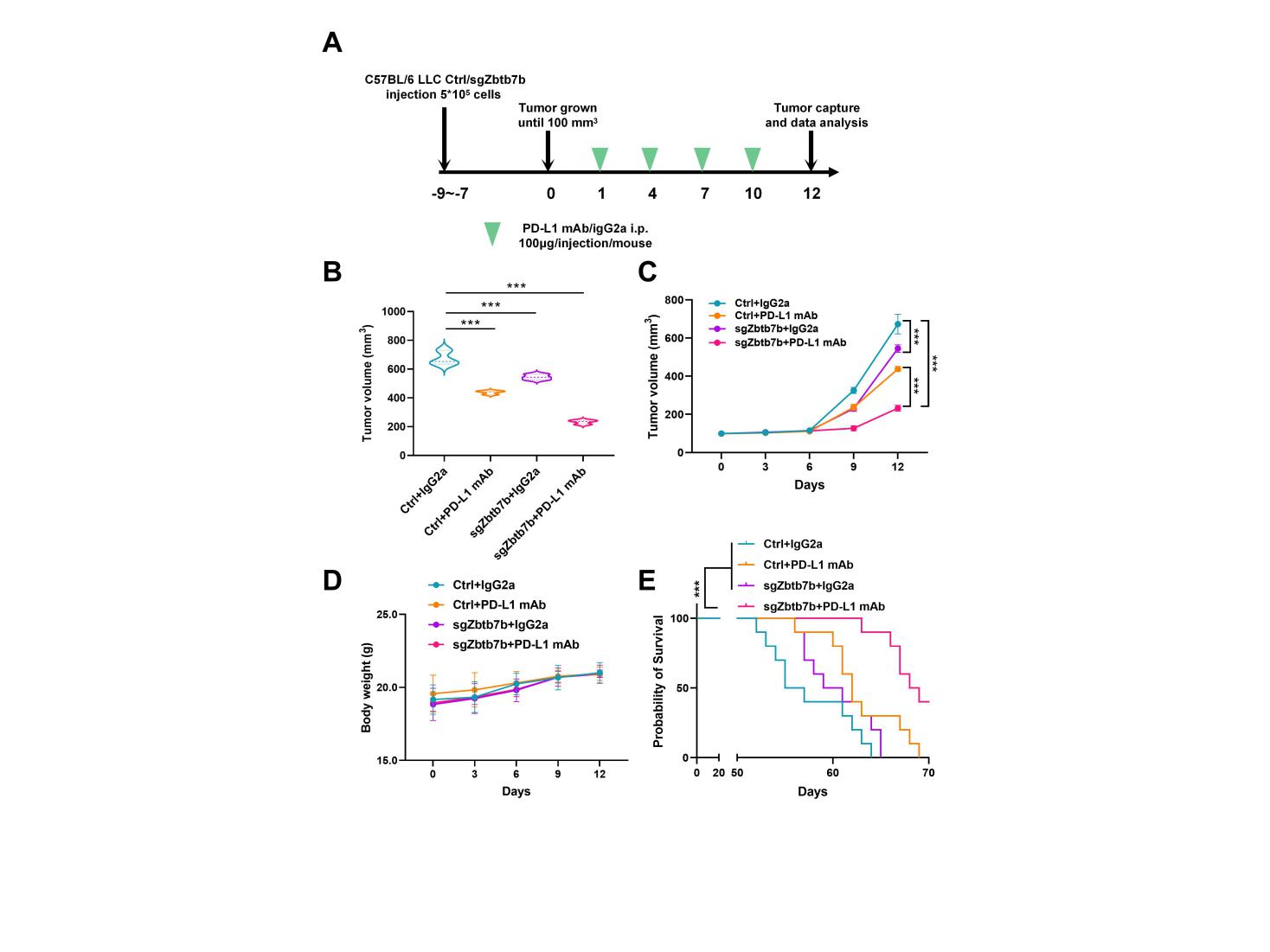
**

**Figure S16** (A-E) BALB/c mice were implanted with 5×10^5^ sgZbtb7b LLC or Ctrl cells and received PD-L1 mAb treatment or IgG isotype control (IgG2a). (A) Schematic diagram of the treatment timeline. (B) Volume statistics of LLC tumors harvested after euthanasia of BALB/c xenografted mice. (C) Line graph of tumor volume over time. (D) Mouse body weight was measured every 3 days. (E) Kaplan-Meier survival curves for each group. Results were presented as mean ± S.D., n = 5. ****P* < 0.001.


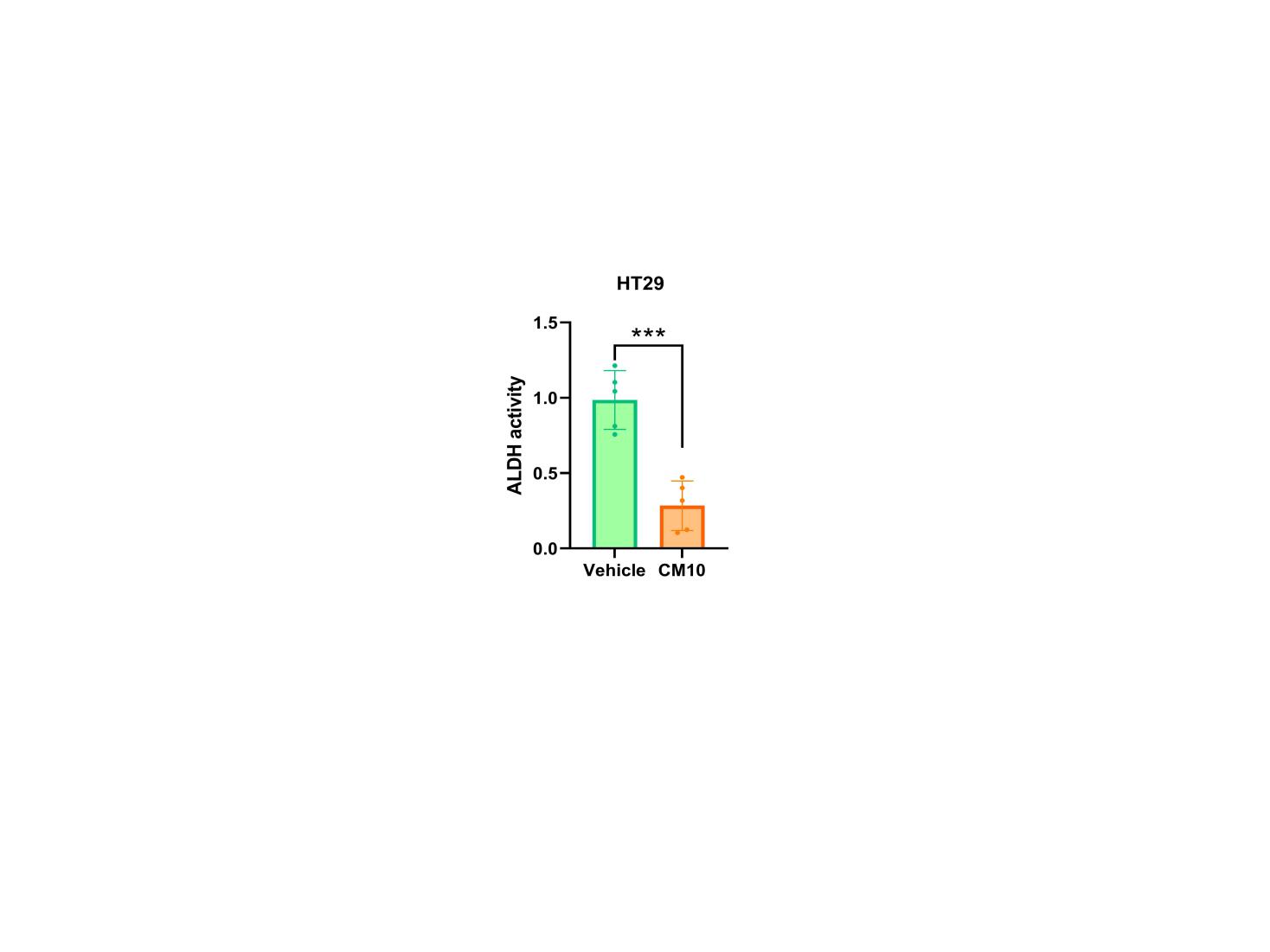


**Figure S17** Bar chart of ALDH enzyme activity after CM10 (10 μM) treatment of HT29 cells for 2 hours. Results were presented as mean ± S.D., n = 5. ****P* < 0.001.


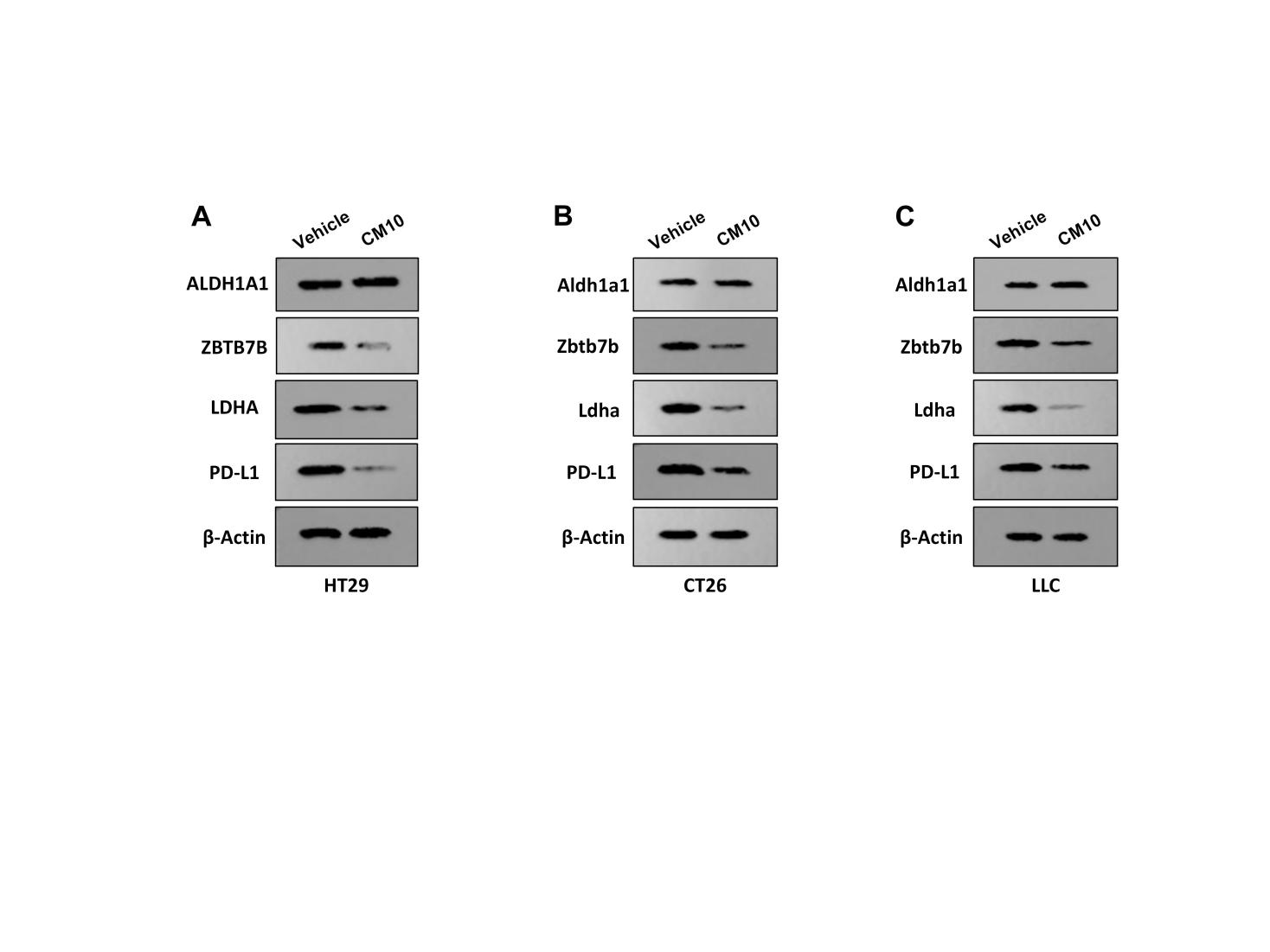


**Figure S18** After CM10 (10 μM) treated HT29 (A), CT26 (B) and LLC (C) cells for 2 hours, western blot detected the expression of ALDH1A1, ZBTB7B, LDHA and PD-L1.

**
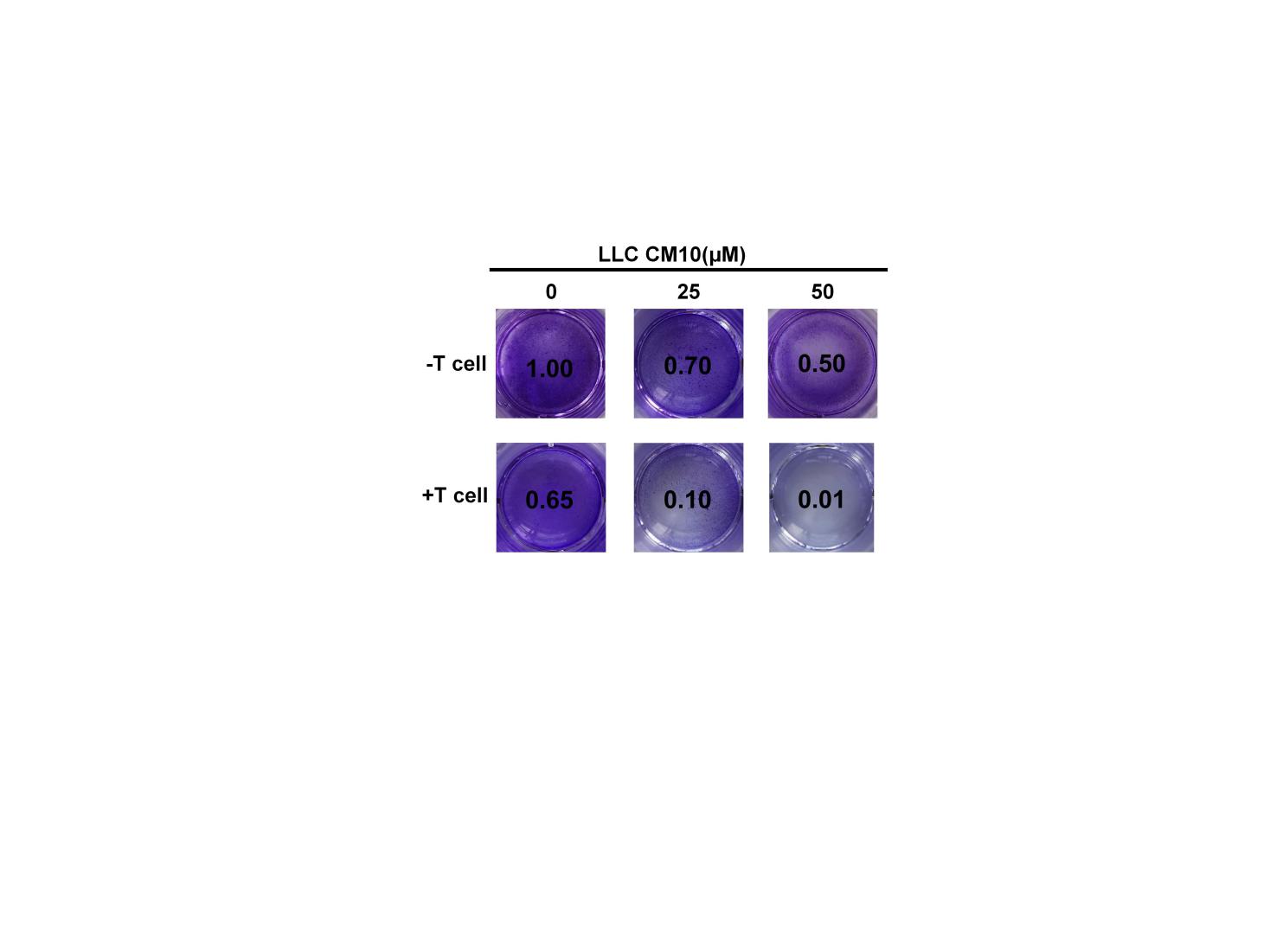
**

**Figure S19** T cell killing assay of LLC cells under different concentrations of CM10 (0, 25, 50 μM).

**
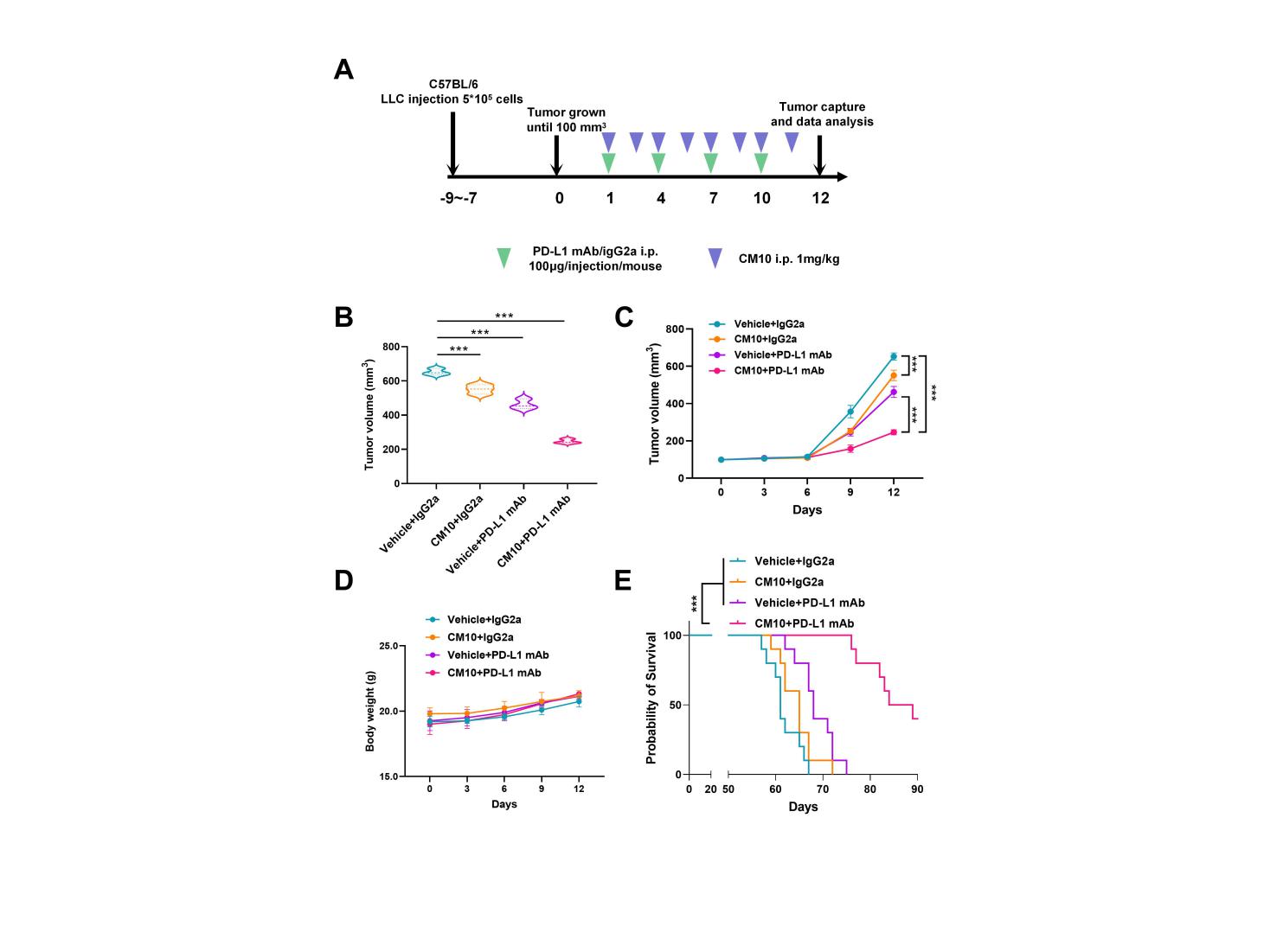
**

**Figure S20** BALB/c mice were implanted with 5×10^5^ LLC cells and treated with CM10 or PD-1 mAb. (A) Schematic diagram of the treatment timeline. (B) Volume statistics of LLC tumors harvested after euthanasia of BALB/c xenografted mice. (C) Line graph of tumor volume over time

(D) Mouse body weight was measured every 3 days. (E) Kaplan-Meier survival curves for each group. Results were presented as mean ± S.D., n = 5. ****P* < 0.001.

**
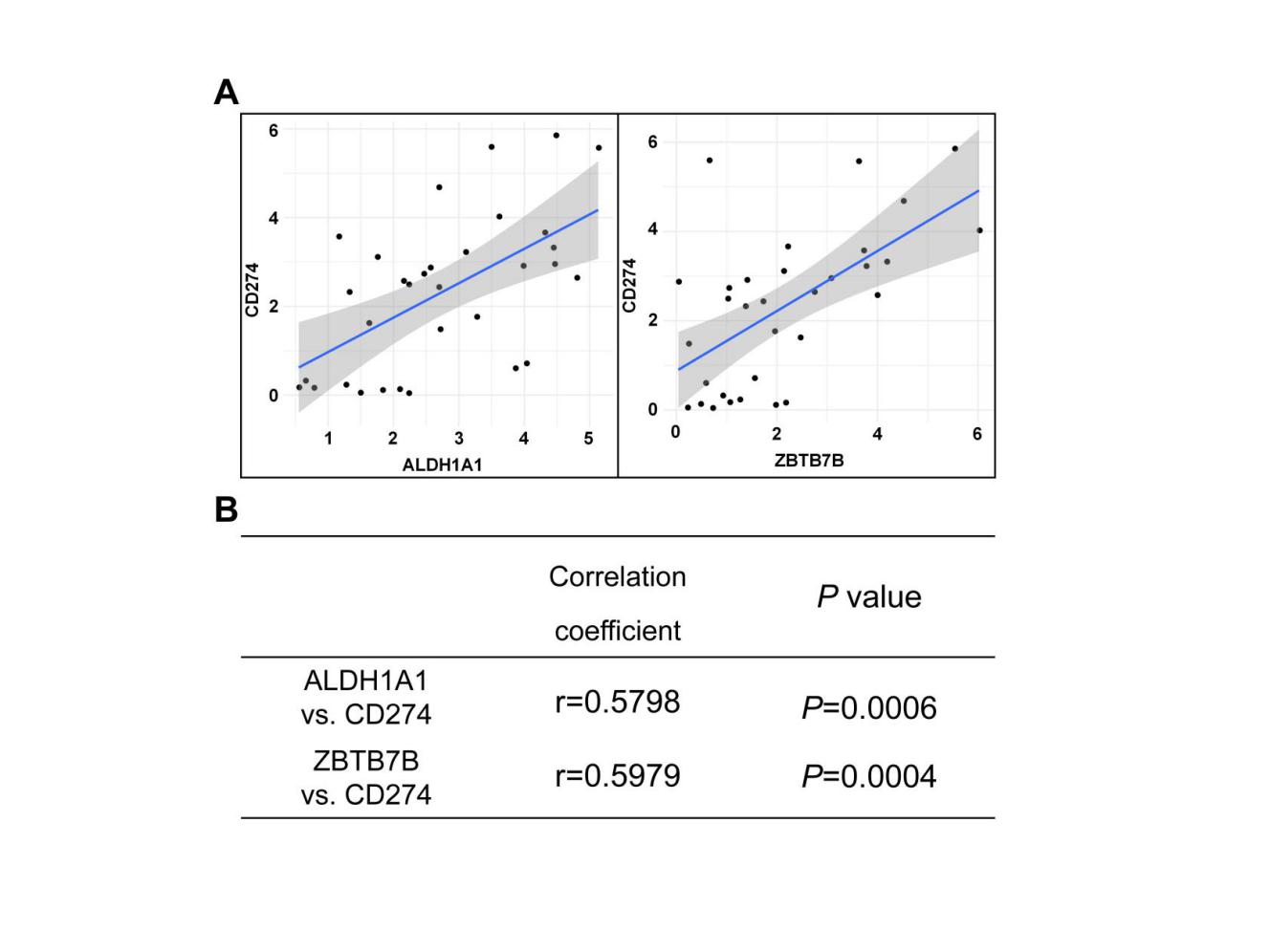
**

**Figure S21** Correlation between expression of ALDH1A1, ZBTB7B and CD274 detected by qPCR in clinical colon cancer tissue. (A) Scatter plot of the correlation between ALDH1A1 and CD274 expression. (B) Scatter plot of the correlation between ZBTB7B and CD274 expression. (C) Quantitative correlation between the expression levels of ALDH1A1, ZBTB7B, and CD274. Correlation coefficients r and *P* values were calculated based on Spearman's rank correlation method.

**
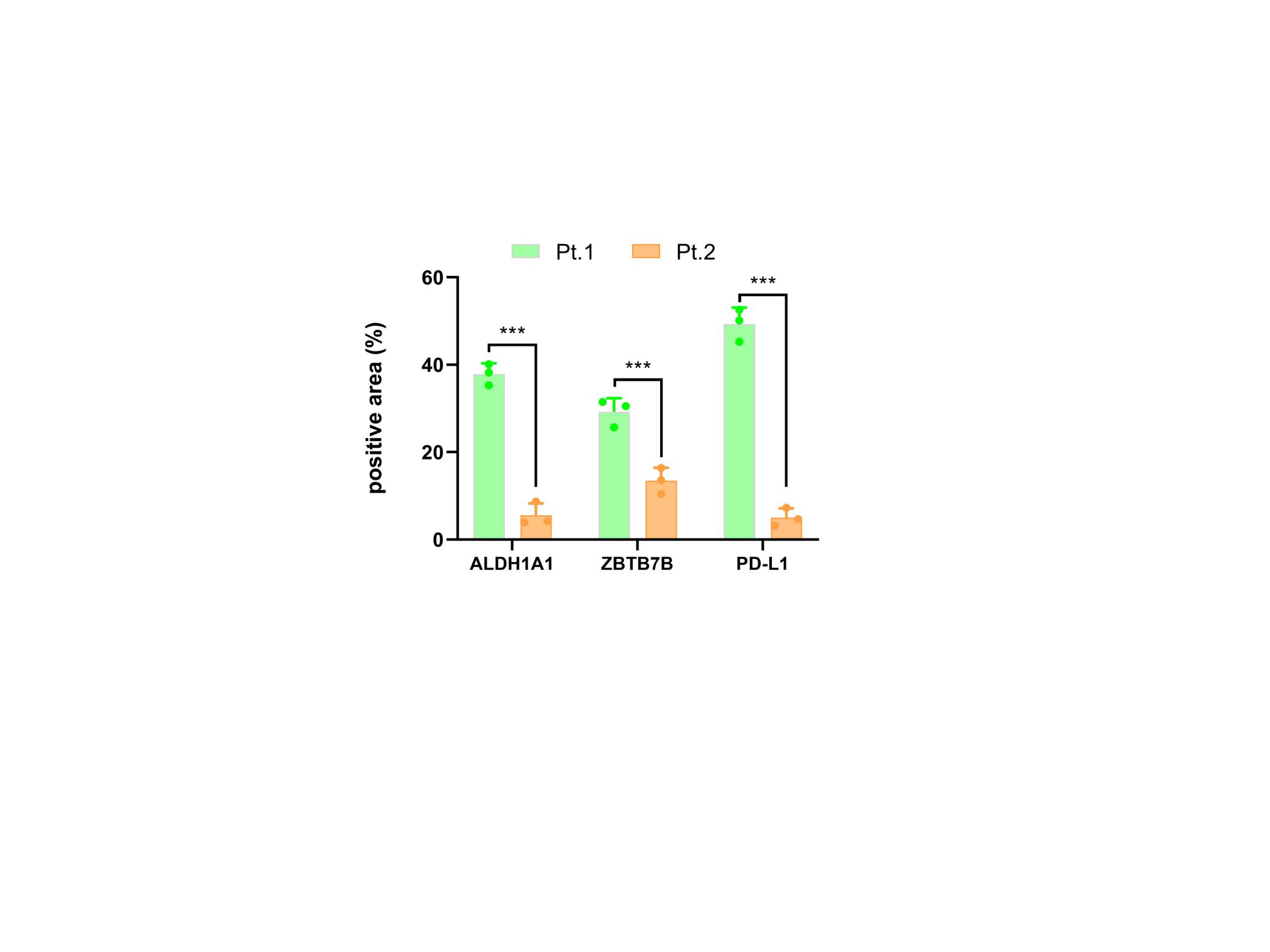
**

**Figure S22** Quantitative statistical histogram of immunohistochemistry in Figure 7C. Results were presented as mean ± S.D., n = 3. ****P* < 0.001.

**Table S1 primer sequences for qPCR.**

| Symbol | Species | Forward (5' -> 3') | Reverse (5' -> 3') |
| --- | --- | --- | --- |
| Xbp1 | Mouse | AGCTTTTACGGGAGAAAACTCAC | CCTCTGGAACCTCGTCAGGA |
| Snrnp70 | Mouse | CCCGAGATCCCATCCCATAC | CAATGCCACAGTAAGGTTGGTTA |
| Tsc22d3 | Mouse | ACCACCTGATGTACGCTGTG | CAGCCGGGACTGGAACTTT |
| Nfe2l2 | Mouse | CTTTAGTCAGCGACAGAAGGAC | AGGCATCTTGTTTGGGAATGTG |
| Hmg20b | Mouse | CACGGGGCCTTTGTAGTGG | TCAGGAAGCGAACATAGCCAG |
| Gata6 | Mouse | TTGCTCCGGTAACAGCAGTG | GTGGTCGCTTGTGTAGAAGGA |
| Gata4 | Mouse | CACCCCAATCTCGATATGTTTGA | GCACAGGTAGTGTCCCGTC |
| Smyd2 | Mouse | ACTGCGACGTGGAATGTCAG | CGCACAGTCTCCGAAGGAT |
| Camta2 | Mouse | GGTTGCTGAGAATAGCCACCA | GACAGCCACTCATCGTGTTTC |
| Id3 | Mouse | CTGTCGGAACGTAGCCTGG | GTGGTTCATGTCGTCCAAGAG |
| Zbtb7b | Mouse | CCCGAGGATGACCTGATTGG | CCTGCGTCCTGATGGTGAG |
| Csde1 | Mouse | CAGCACTTCGTGAAACTGGG | TAGCTTGCCGTTCTGAACACT |
| Srebf2 | Mouse | GCAGCAACGGGACCATTCT | CCCCATGACTAAGTCCTTCAACT |
| Taf7 | Mouse | GCCAGTTTATCTTACGACTGCC | CATCAGGGTGTAACTCAATGCTC |
| Dido1 | Mouse | GAAGCACCCAAGGCTATCAAA | GCGCAGGGAGAGGTTATGC |
| LDHA | Human | ATGGCAACTCTAAAGGATCAGC | CCAACCCCAACAACTGTAATCT |
| Ldha | Mouse | CAAAGACTACTGTGTAACTGCGA | TGGACTGTACTTGACAATGTTGG |
| ALDH1A1 | Human | GCACGCCAGACTTACCTGTC | CCTCCTCAGTTGCAGGATTAAAG |
| ZBTB7B | Human | CCTCCTCAGTTGCAGGATTAAAG | AGCTTAGGTAGGCCATCAGGT |
| CD274 | Human | TGGCATTTGCTGAACGCATTT | TGCAGCCAGGTCTAATTGTTTT |

**Table S2 summary of top20 differentially expressed transcription factor genes between shALDH1A1 and CTRL HT29 cells.**

| Genes | logFC | P.Value |
| --- | --- | --- |
| XBP1 | -4.872034828 | 0.000892762 |
| SNRNP70 | -4.781413472 | 0.014094381 |
| TSC22D3 | -4.758181064 | 0.000291976 |
| NFE2L2 | -4.624909586 | 7.34796E-05 |
| HMG20B | -4.390434935 | 0.000112515 |
| GATA6 | -4.380141876 | 0.000977798 |
| GATA4 | -4.317710597 | 0.007003246 |
| SMYD2 | -4.263232729 | 0.005749717 |
| CAMTA2 | -4.201305946 | 0.013300913 |
| ID3 | -4.18333061 | 0.000317922 |
| **ZBTB7B** | -4.170974535 | 0.008820149 |
| CSDE1 | -4.133689663 | 0.003226322 |
| SREBF2 | -4.102962133 | 0.001699824 |
| TAF7 | -4.011881284 | 0.011634985 |
| DIDO1 | -4.00150777 | 0.002603614 |
| PSMB1 | -3.983577806 | 0.002415098 |
| STAG2 | -3.974214807 | 0.001749671 |
| CHD3 | -3.948359432 | 0.000299163 |
| CREBL2 | -3.939653327 | 0.000704036 |

**Table S3 Clinicopathologic characteristics of PD-1 mAb monotherapy cohort.**

| **Patient No.** | **Gender** | **Age** | **Tumor types** | **TNM stage** | **Response** | **PS score** | **PFS (months)** | **Adverse effect** | **Metastatic lesion** |
| --- | --- | --- | --- | --- | --- | --- | --- | --- | --- |
| 1 | Female | 74 | LUAD | T1N2M1b | SD | 1 | 12+ | None | brain |
| 2 | Male | 80 | LUAD | T1N2M1b | SD | 1 | 9+ | None | liver |
| 3 | Male | 53 | LUAD | T1N2M1c | PR | 1 | 24+ | None | brain,adrenal glands |
| 4 | Male | 56 | LUSC | T1N2M1b | PR | 1 | 17+ | cough,vomit | bone |
| 5 | Male | 74 | LUAD | T1N2M1a | PR | 1 | 10+ | cough | pericardial effusion |
| 6 | Male | 74 | LUAD | T1NxM1c | PD | 1 | 11 | vomit | liver,brain |
| 7 | Male | 70 | LUSC | T3N3M1a | PD | 1 | 5 | cough | pericardial effusion |
| 8 | male | 60 | LUAD | T2N3M1a | PR | 1 | 6+ | None | pleural nodules |
| 9 | Male | 60 | LUAD | T4N2M1b | PR | 1 | 13+ | None | bone |
| 10 | Male | 66 | LUSC | T3N1M1b | PR | 1 | 15+ | None | bone |
| 11 | Male | 60 | LUAD | T1N3M1b | PR | 1 | 20+ | vomit | bone |
| 12 | Male | 58 | LUAD | T3N0M1b | SD | 1 | 12+ | None | brain |
| 13 | Male | 60 | LUSC | T2N2M1b | PD | 1 | 12 | None | brain |
| 14 | Male | 62 | LUAD | T3N0M1c | PD | 1 | 7 | cough | brain, liver, bone, adrenal glands |
| 15 | Female | 66 | LUAD | T2N1M1b | PD | 1 | 8 | vomit | brain |
| 16 | Male | 60 | LUSC | T2N2M1c | SD | 1 | 6+ | cough | liver,bone |
| 17 | Female | 72 | LUAD | T2N2M1b | PD | 1 | 9 | erythra, vomit | bone |
| 18 | Male | 56 | LUSC | T3N2M1c | PD | 1 | 3 | vomit | mediastinum, neck |
| 19 | Male | 64 | LUSC | T3N2M1b | PD | 1 | 9 | cough | bone |
| 20 | Male | 73 | LUAD | T1N1M1b | PD | 1 | 6 | cough,vomit | brain |

PD-1, programmed death-1; PR, partial response; SD, stable disease; PD, progressive disease; PFS, progression-free survival. Patients were stratified into response groups based on RECIST 1.1 criteria. Patients with PR and SD > 3 months were classified as responders, while patients with SD ≤ 3 months and PD were classified as non-responders. LUSC: Lung squamous cell carcinoma. LUAD: Lung adenocarcinoma. TNM stage based on the 8th Edition Lung Cancer Stage Classification.

**Table S4 Clinicopathologic characteristics of PD-1 mAb monotherapy cohort.**

|  | PD-1 mAb Monotherapy | |
| --- | --- | --- |
| Patient Characteristics | Responders (11) | Non-responders (9) |
| Age (years), mean±SE | 63.73±8.23 | 66.34±5.97 |
| Male | 10 | 7 |
| Female | 1 | 2 |
| Response, n (%） |  |  |
| CR | 0 | 0 |
| PR | 7 (63.64) | 0 |
| SD | 4(36.36) | 0 |
| PD | 0 | 9(100) |
| Median PFS (months) | 13.09±5.35 | 7.78±2.70 |
| 12 months PFS (%) | 63.64 | 11.11 |

PD-1, programmed death-1; CR, complete response; PR, partial response; SD, stable disease; PD, progressive disease; PFS, progression-free survival. Patients were stratified into response groups based on RECIST 1.1 criteria. Patients with CR, PR, and SD > 3 months were classified as responders, while patients with SD ≤ 3 months and PD were classified as non-responders.
