# Supplementary material for: ALDH1A1 promotes immune escape of tumor cells through ZBTB7B-glycolysis pathway
Source: Cell Death Dis. 2024 Aug 7;15(8):568. doi: 10.1038/s41419-024-06943-9 (PMC11303523; doi:10.1038/s41419-024-06943-9)
Supplement: Supplementary file 2 — Uncropped WB images [file 41419_2024_6943_MOESM2_ESM.pdf]

Figure 1L

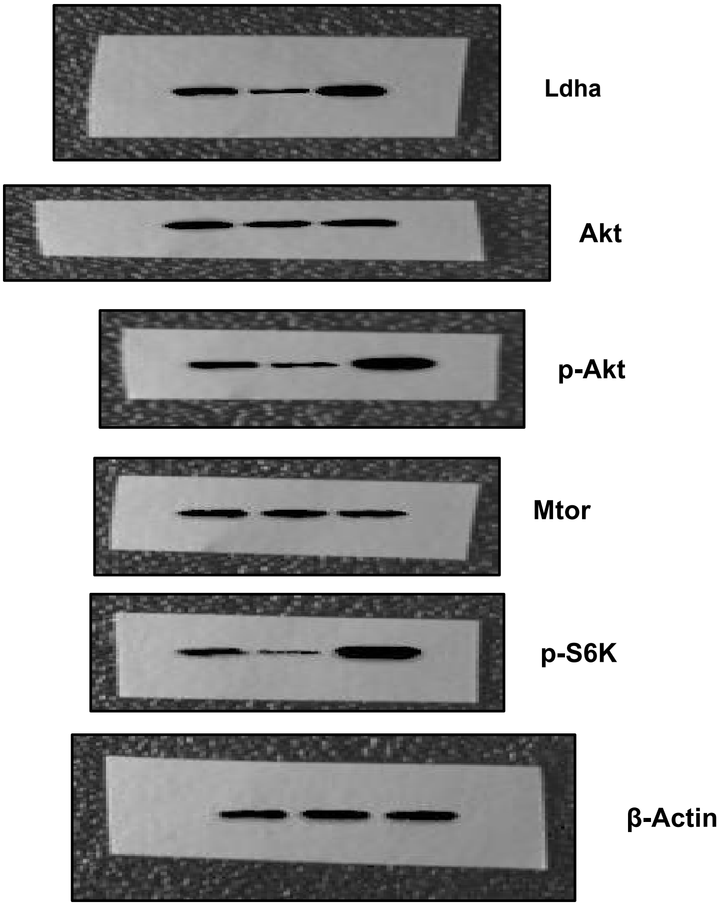

Figure 4D

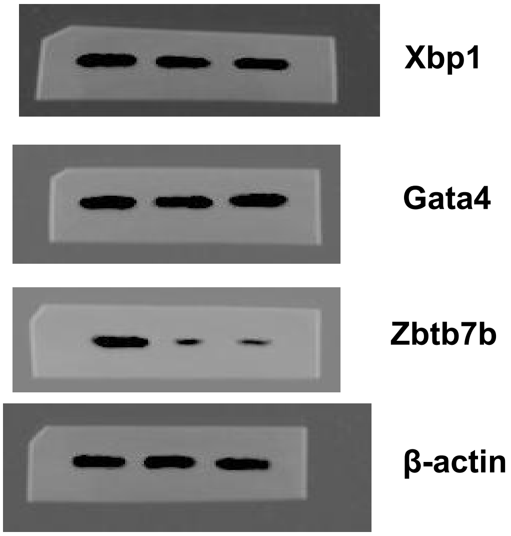

Figure 4J

Figure 4E

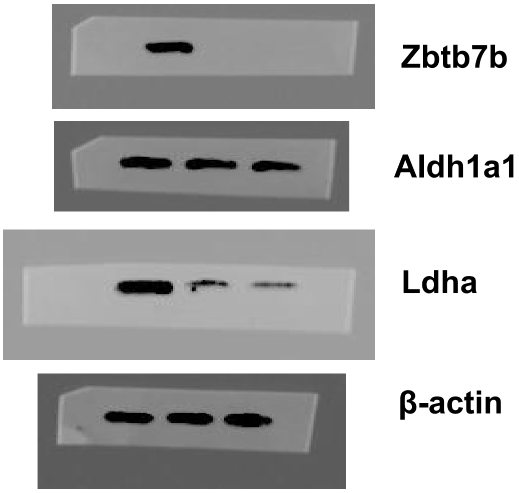

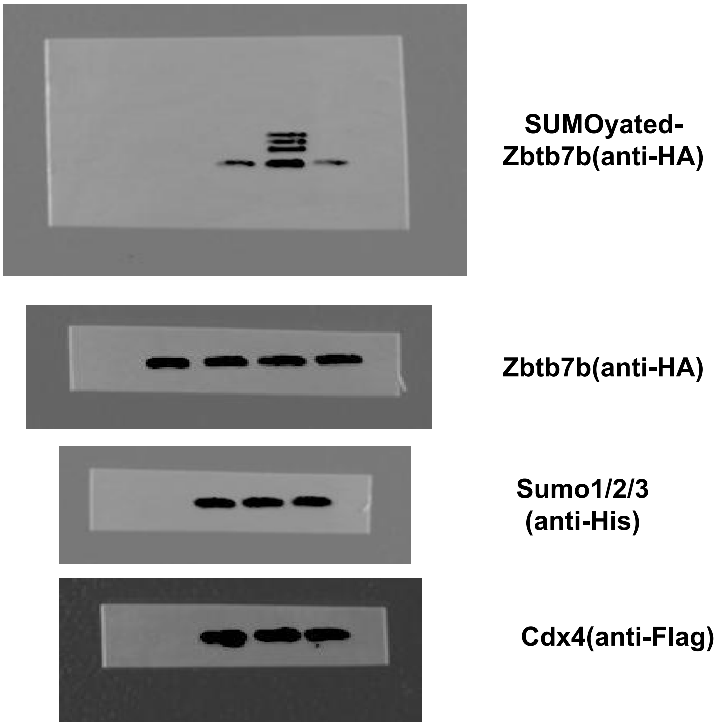

Figure 5D

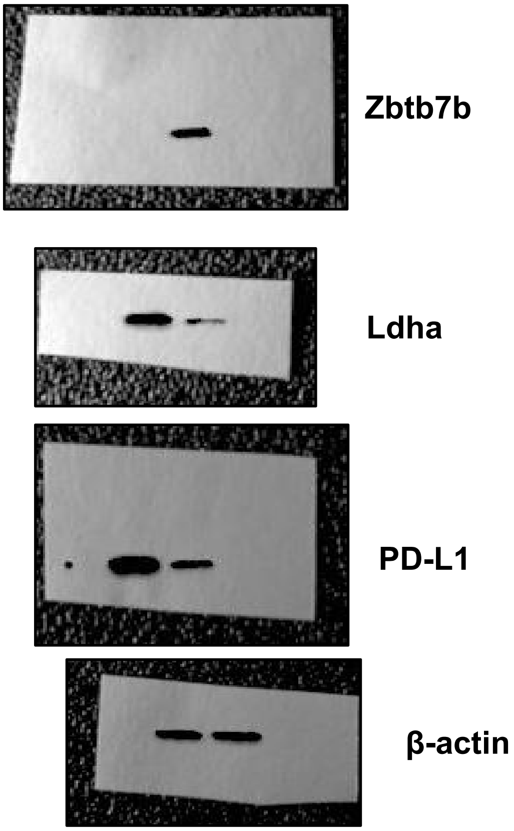

Figure S1A

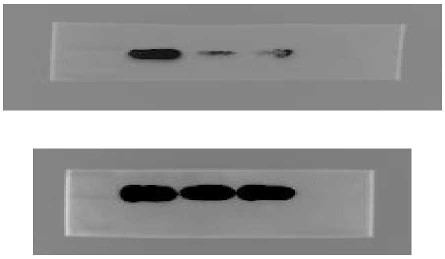

ALDH1A1

β-Actin

Figure S1B

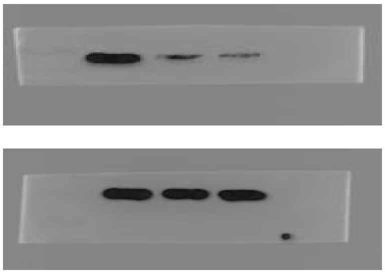

ALDH1A1

β-Actin

Figure S1C

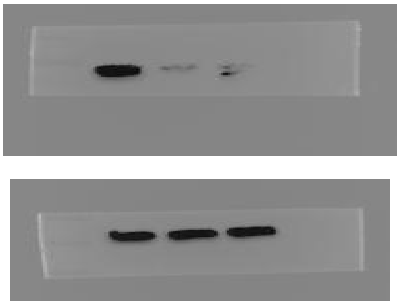

Aldh1a1

β-Actin

Figure S6B

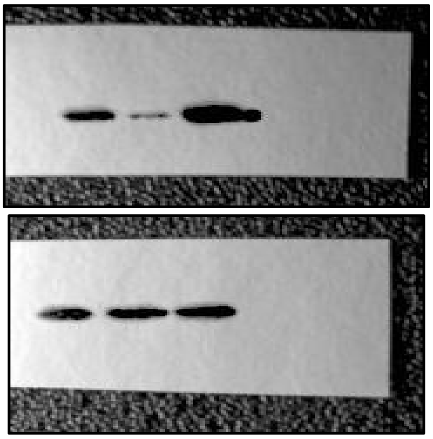

LDHA

β-Actin

Figure S6C

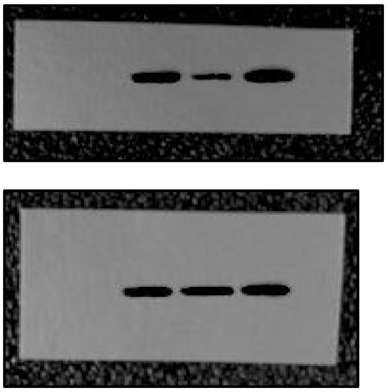

LDHA

β-Actin

Figure S7B

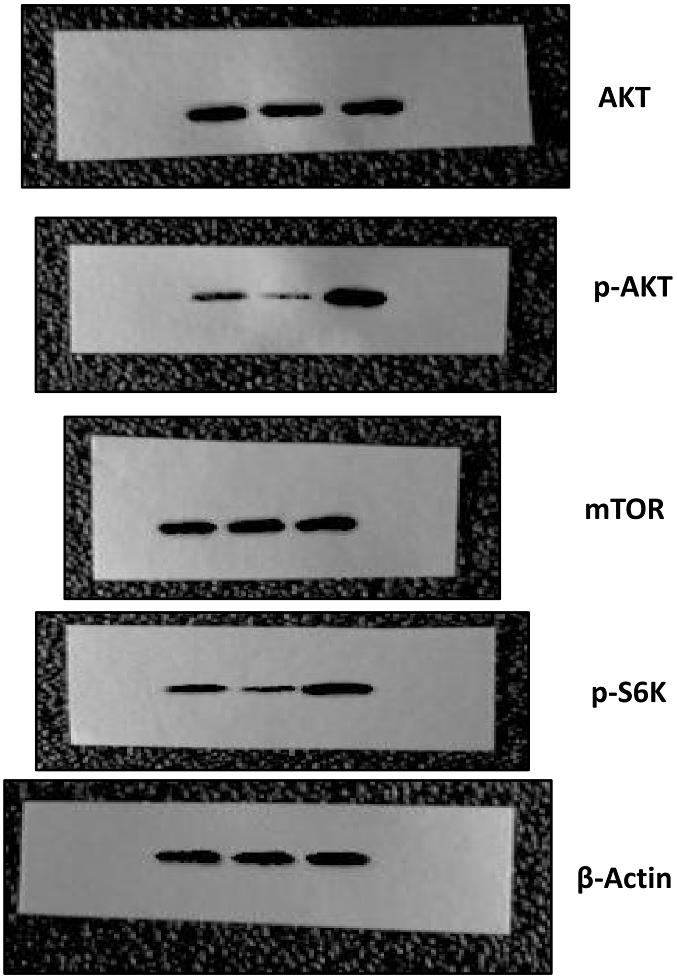

Figure S7C

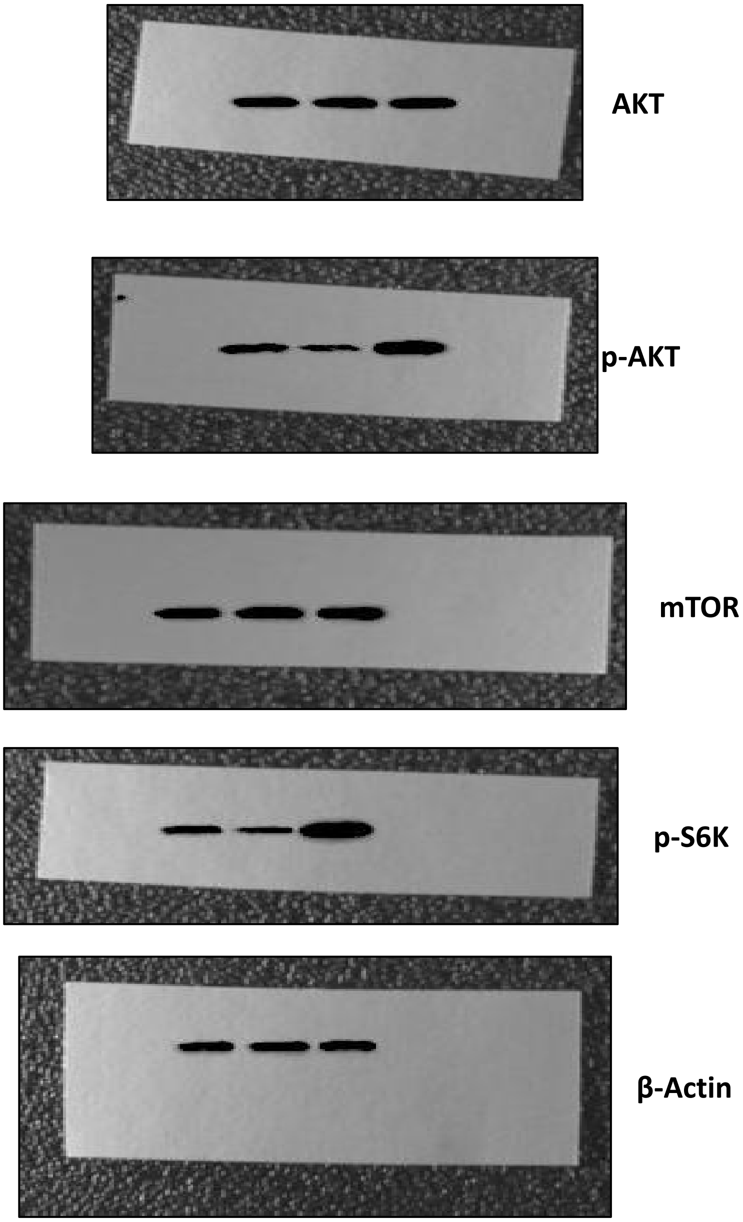

Figure S13

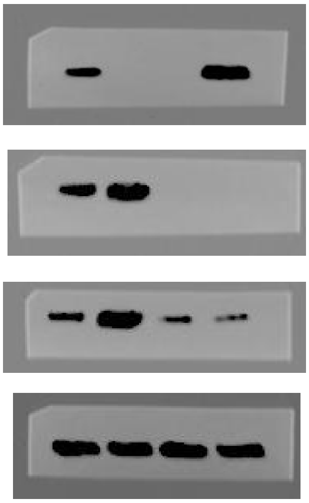

Figure S14A

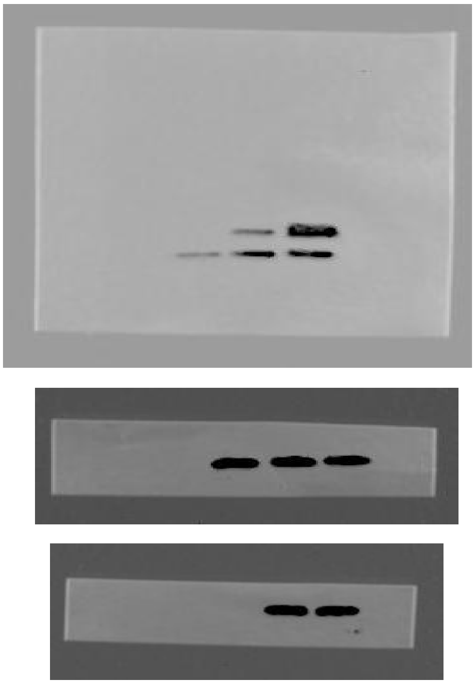

Figure S14B

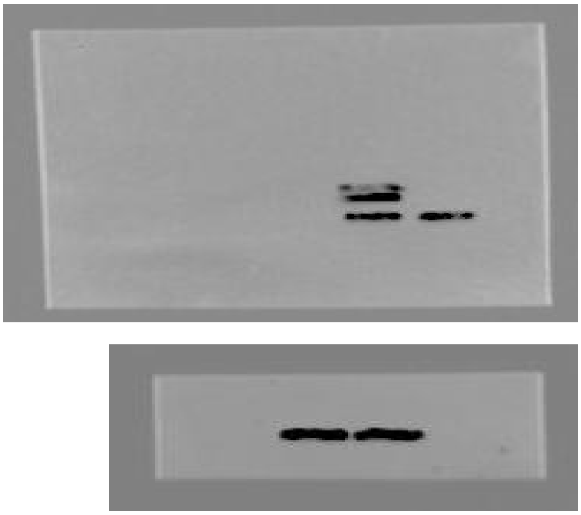

Figure S15B

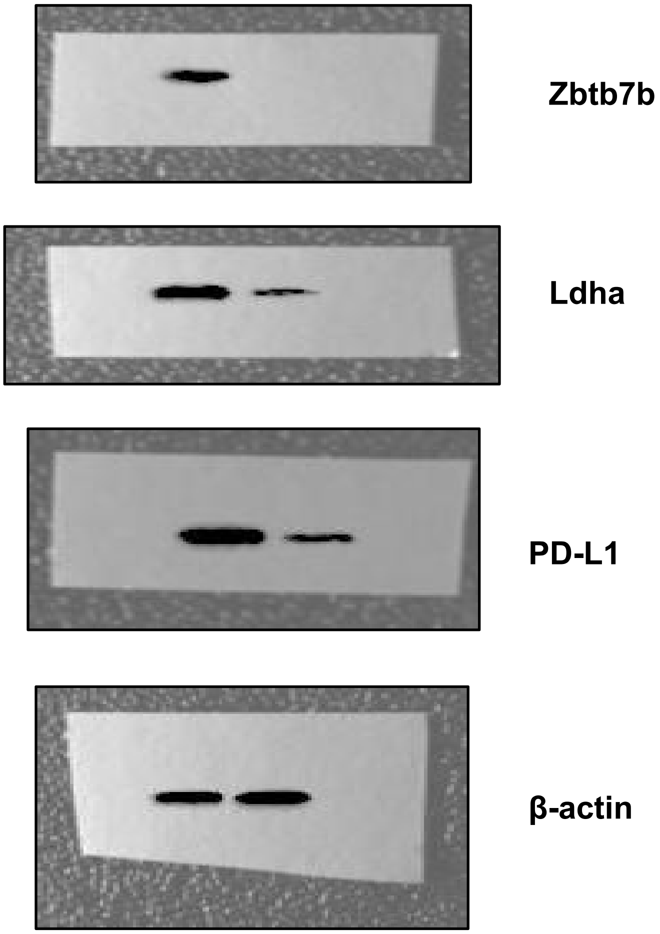

Figure S18

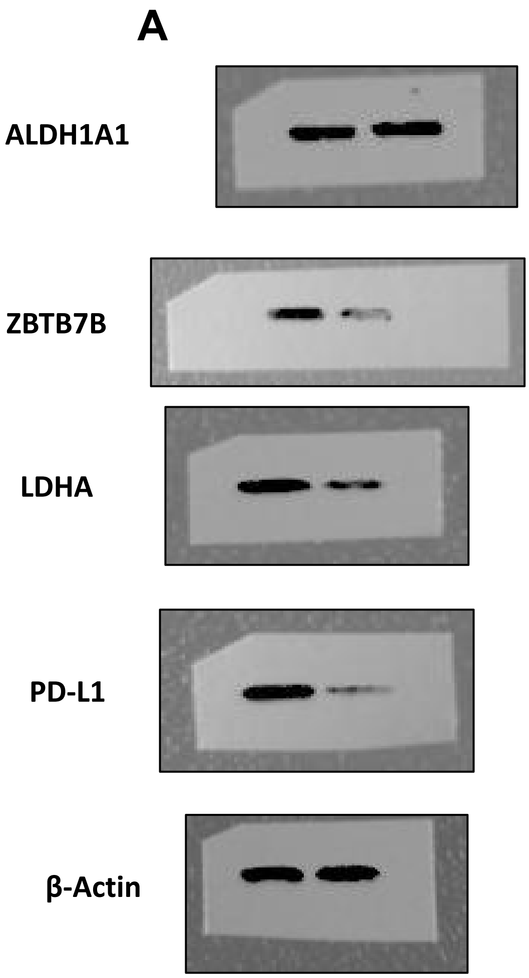
A
ALDH1A1
ZBTB7B
LDHA
PD-L1
β-Actin

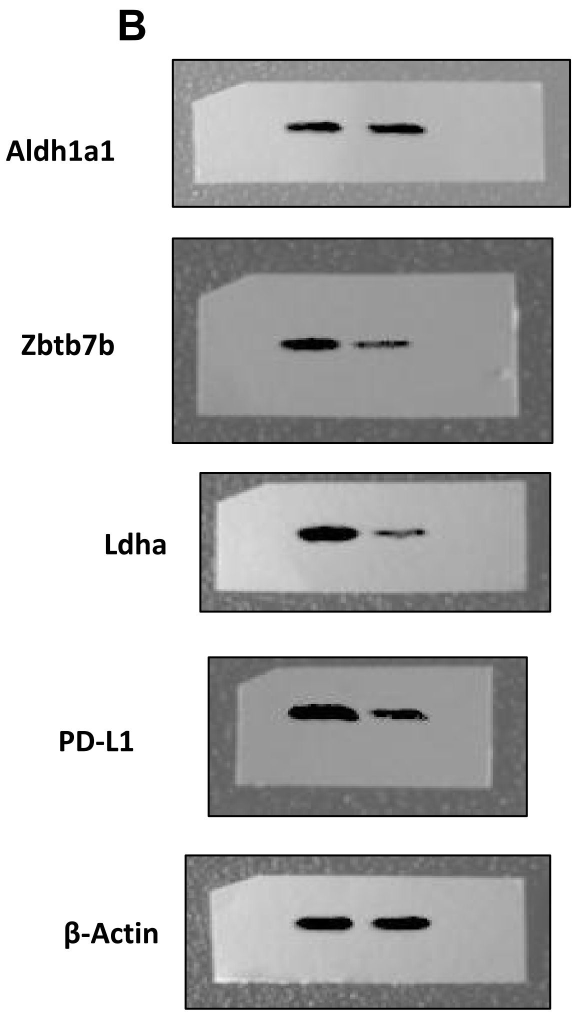
B
Aldh1a1
Zbtb7b
Ldha
PD-L1
β-Actin

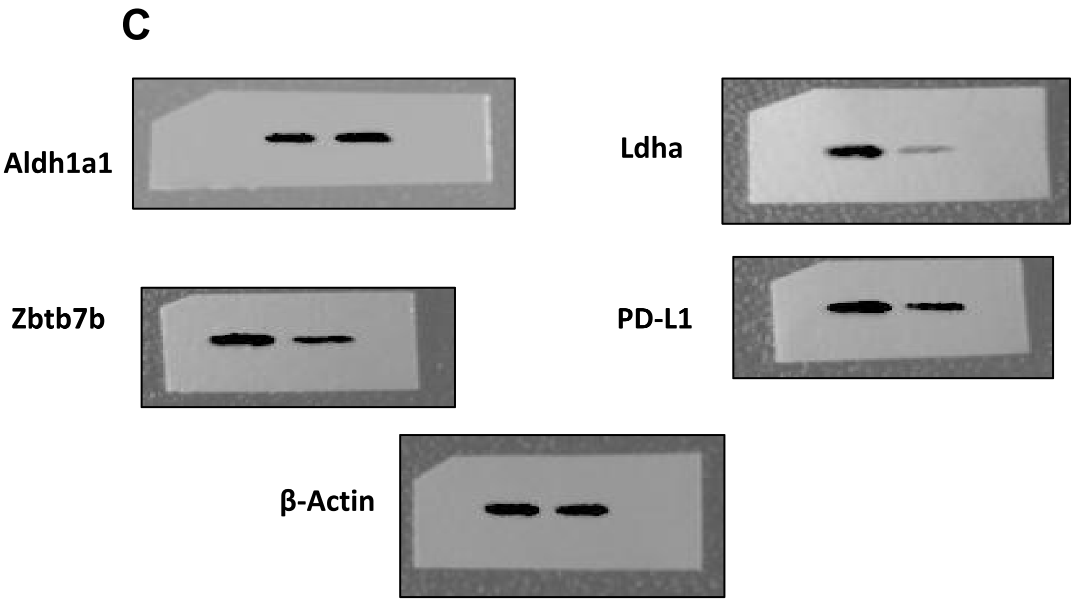
C
Aldh1a1
Ldha
Zbtb7b
PD-L1
β-Actin
